# Integrative Transcriptomic and microRNAomic Profiling Reveals Immune Mechanism for the Resilience to Soybean Meal Stress in Fish Gut and Liver

**DOI:** 10.3389/fphys.2018.01154

**Published:** 2018-09-10

**Authors:** Nan Wu, Biao Wang, Zheng-Wei Cui, Xiang-Yang Zhang, Ying-Yin Cheng, Xuan Xu, Xian-Mei Li, Zhao-Xi Wang, Dan-Dan Chen, Yong-An Zhang

**Affiliations:** ^1^State Key Laboratory of Freshwater Ecology and Biotechnology, Institute of Hydrobiology, Chinese Academy of Sciences, Wuhan, China; ^2^Laboratory for Marine Biology and Biotechnology, Qingdao National Laboratory for Marine Science and Technology, Qingdao, China; ^3^Key Laboratory of Aquaculture Disease Control, Ministry of Agriculture, Wuhan, China; ^4^College of Modern Agriculture Sciences, University of Chinese Academy of Sciences, Beijing, China; ^5^Hubei Key Laboratory of Agricultural Bioinformatics, College of Informatics, Huazhong Agricultural University, Wuhan, China

**Keywords:** soybean meal induced enteritis, resilience, transcriptome, miRNAome, gut-liver immunity, grass carp

## Abstract

In aquafeeds, fish-meal has been commonly replaced with plant protein, which often causes enteritis. Currently, foodborne enteritis has few solutions in regards to prevention or cures. The recovery mechanism from enteritis in herbivorous fish may further help understand prevention or therapy. However, few reports could be found regarding the recovery or resilience to fish foodborne enteritis. In this study, grass carp was used as an animal model for soybean meal induced enteritis and it was found that the fish could adapt to the soybean meal at a moderate level of substitution. Resilience to soybean meal stress was found in the 40% soybean meal group for juvenile fish at growth performance, morphological and gene expression levels, after a 7-week feeding trial. Furthermore, the intestinal transcriptomic data, including transcriptome and miRNAome, was applied to demonstrate resilience mechanisms. The result of this study revealed that in juvenile grass carp after a 7-week feeding cycle with 40% soybean meal, the intestine recovered via enhancing both an immune tolerance and wound healing, the liver gradually adapted via re-balancing immune responses, such as phagosome and complement cascades. Also, many immune factors in the gut and liver were systemically revealed among stages of on-setting, remising, and recovering (or relief). In addition, miRNA regulation played a key role in switching immune states. Thus, the present data systemically demonstrated that the molecular adaptation mechanism of fish gut-liver immunity is involved in the resilience to soybean meal stress.

## Introduction

With the rapid expansion of the fish farming industry and the limited availability of catching wild fish, the global contribution of fish-meal (FM) to aquafeeds has dramatically decreased (Krol et al., 2016). As a result, FM has been replaced by plant proteins, such as soybean meal (SBM), which contains many anti-nutritional factors. Consequently, plant protein induced fish enteritis has been one of the major challenges for sustainable aquaculture (Hu et al., 2016), and this occurs in a dose-dependent manner (De Santis et al., 2015; Krol et al., 2016).

In fish foodborne enteritis, such as soybean meal induced enteritis (SBMIE), the immunity of both the gut and liver have been revealed to be strongly disturbed (De Santis et al., 2015). The gut-liver immunity has been suggested to be not only in mammals (Trivedi and Adams, 2016), but also in fish (Wu et al., 2016). This was based on many different immune processes that were revealed by several systemic studies of both the gut and liver (De Santis et al., 2015; Wu et al., 2016). The intestinal mucosal surface plays an important role as the first line of defense, and intestinal immune cells and molecules are involved in either maintaining or restoring immune homeostasis (Nowarski et al., 2017). Fish gut contains many adaptive immune factors, whereas hepatic tissue contains many innate factors, and also secretes bile, containing a lot of direct immune effectors. The liver functions as a secondary “firewall” and protects the body from intestinal food antigens crossing the intestinal barrier (Brandl et al., 2017). Thus, a study on both the gut and liver is important to reveal the mechanism involved in nutritional stress caused by SBM.

Recently, in Atlantic salmon (*Salmo salar*), transcriptomic studies provided insights into foodborne enteritis (Tacchi et al., 2012; De Santis et al., 2015; Krol et al., 2016). The intestinal response to SBM in salmonids is possibly a lymphocyte-mediated and lymphokine-driven inflammation (Romarheim et al., 2011). The pro-inflammatory cytokine IL17 (Marjara et al., 2012) and anti-inflammatory cytokine IL10 (Torrecillas et al., 2015) play an important role in SBMIE. Further, complement cascades in the liver as well as phagocytosis in the distal intestine (DI), have been revealed as the most important factor for SBM stress (De Santis et al., 2015). Transcriptomic differences between marine and plant diet groups in the intestine and liver were revealed in Atlantic salmon, without any histopathological signs (Tacchi et al., 2012). In the plant diet group, higher levels of gene expression were involved in enteritis, protein and energy metabolism, mitochondrial activity/kinases and transport. Lower gene expression was involved in cell proliferation and apoptosis, this was observed in the DI. In the liver, a lower expression of immune response genes was found but there was a higher expression of genes involved in cell proliferation and apoptosis processes.

As an important aspect at a transcriptional level, regulation of mRNA expression by miRNA also exists in fish. Fish diet could affect the expression of miRNAs and their targets (Mennigen et al., 2012; Craig et al., 2014; Geurden et al., 2014; Bizuayehu et al., 2016; Miao et al., 2017). For example, different foods eaten during the initial feeding changed the miRNA and their targets (Bizuayehu et al., 2016) in Atlantic cod (*Gadus morhua*) larvae. In trout alevins, a short hyperglucidic–hypoproteic stimulus had a long-term influence on muscle glucose metabolism and intestinal microbiota via miRNA regulation (Geurden et al., 2014). For hepatic miRNAs, negative regulation of hepatic insulin signaling in rainbow trout (*Oncorhynchus mykiss*) (Mennigen et al., 2012), and the disturbance of key hepatic metabolic pathways during a fasting response in zebrafish (*Danio rerio*) (Craig et al., 2014), has been revealed. Other reports also showed that both hepatic and intestinal miRNAs were involved in glucose metabolism during a high-starch diet feeding in blunt snout bream (*Megalobrama amblycephala*) (Miao et al., 2017).

However, in order to gain information about prevention or therapy, attention should be paid to the recovery or resilience of fish SBMIE, as there have been few reports recently. In carnivorous fish, such as the Atlantic salmon, a low inclusion of SBM in their diet of about 12% was safe (Sanden et al., 2005), whereas high SBM dietary inclusion levels, such as at 30%, could cause intestinal inflammation (Sealey et al., 2015). Other reports suggest that there was no sign of recovery even in a low dosage (10%) of SBM feeding in salmon (Uran et al., 2009). However, in European sea bass (*Dicentrarchus labrax*), an omnivorous fish, SBM of up to 30% can be successfully incorporated into feeds containing low FM inclusion (Bonvini et al., 2018). In common carp (*Cyprinus carpio L*.), after 5-weeks of continuous feeding with 20% SBM, the recovery from SBMIE was assessed and it was found that most of the assessed parameters (expression levels of cytokines) appeared to return to around normal levels, yet the supranuclear vacuoles (SNV) did not appear to be the normal size and the lamina propria (LP) was still thicker (Uran et al., 2008). These findings suggest that a certain degree of resilience to SBM stress possibly exists, at least in omnivorous fish and speculatively in herbivorous fish.

Grass carp (*Ctenopharyngodon idella*), as a typical herbivorous fish, may easily accept plant protein and be more able to develop resilience. However, grass carp intestinal immunity could also be influenced by other factors on the plant proteins (Xu et al., 2016; Zhang et al., 2017). Therefore, in this study, grass carp were used to examine the resilience mechanisms used during SBM stress. Integrative analysis of intestinal transcriptome, miRNAome, and hepatic transcriptome, were completed in the 7-week feeding trial, by paralleling a partially substituted (40% SBM, 40SBM) diet, with controls [100% FM as a negative, while 70SBM (70% SBM) as a positive]. The results demonstrated that during the feeding trial, the DI and liver tissues gradually developed their own adaptation strategies, respectively. Upon 40SBM feeding, acute intestinal inflammation was found in the early stages, and was then relieved by both tolerance and wound healing. In the liver, increased oil droplets and inflammation cytokines were found at first and then followed an adjusted immune reaction, such as possible working of certain macrophage and T cell population as well as alternative complement pathway activating, finally maintained the homeostasis. Additionally, intestinal miRNA regulation played an important role in transcriptional adjustment during resilience. Thus, the current study sheds some lights on immune mechanisms for the resilience to SBM stress in fish gut and liver.

## Materials and methods

### Animals and diets

Grass carp were used as a herbivorous fish. The use of animals in this study was approved by the Animal Research and Ethics Committees of the Institute of Hydrobiology, Chinese Academy of Sciences. All experiments were conducted in accordance with the guidelines of the committees. The formulation of the experimental diets are shown in Table 1. Diets were formulated to be iso-nitrogenous and isoenergetic on a crude protein (350.0 g/kg) and a crude lipid (50.0 g/kg) basis according to Ji et al. (2011) and National Research Council (2011). All ingredients were thoroughly mixed with fish oil and soybean oil, and then deionized water was added to produce a stiff dough. The pellets were prepared by extrusion and air-drying. After being prepared completely, the pellets were stored at −20°C.

**Table 1:** Diet formulation and composition.

	**Diet**		
	**FM**	**40SBM**	**70SBM**
**INGREDIENTS**			
Fish meal	450.0	240.0	0.0
Soybean meal	–	400.0	700
Glycine	45.0	0.0	0.0
Fish oil	18.0	18.0	30.0
Soybean oil	20.0	20.0	20.0
Corn starch	337.0	212.0	155.0
Ca (H2PO4)2	24.0	24.0	24.0
Mineral premix^a^	20.0	20.0	20.0
Vitamin premix^b^	10.0	10.0	10.0
Choline chloride (500 g kg^−1^)	6.0	6.0	6.0
Cellulose	70.0	50.0	0.0
Ethoxyquin (300 g kg^−1^)	0.5	0.5	0.5
**Total**	1000.0	1000.0	1000.0

a *Per kilogram of mineral premix (g kg^−1^): MnSO_4_·H_2_O (318 g kg^−1^Mn), 1.640g; MgSO_4_·H_2_O (150 g kg^−1^ Mg), 60.530 g; FeSO_4_·H_2_O (300 g kg^−1^ Fe), 23.110 g; ZnSO_4_·H_2_O (345 g kg^−1^ Zn), 0.620 g; CuSO_4_·5H_2_O (250 g kg^−1^ Cu), 0.010 g; KI (38 g kg^−1^ I), 0.070 g; NaSeO_3_ (10 g kg^−1^ Se), 0.005 g. All ingredients were diluted with corn starch to 1 kg*.

b *Per kilogram of vitamin premix (g kg^−1^): retinyl acetate (500 000 IU g^−1^), 2.40 g; cholecalciferol (500 000 IU g^−1^), 0.40 g; DL-α-tocopherol acetate (500 g kg^−1^), 12.55 g; menadione (230 g kg^−1^), 0.80 g; cyanocobalamin (10 g kg^−1^), 0.83 g; D-biotin (20 g kg^−1^), 4.91 g; folic acid (960 g kg^−1^), 0.40 g; thiamin hydrochloride (980 g kg^−1^), 0.05 g; ascorhyl acetate (930 g kg^−1^), 7.16 g; niacin (990 g kg^−1^), 2.24 g; meso-inositol (990 g kg^−1^), 19.39 g; calcium-D-pantothenate (980 g kg^−1^), 2.89 g; riboflavin (800 g kg^−1^), 0.55 g; pyridoxine (980 g kg^−1^), 0.59 g. All ingredients were diluted with corn starch to 1 kg*.

### Feeding trial

The juvenile grass carps were obtained from Shishoulaohe Yangtze River Four Major Chinese Carp Native Species Farm (Hubei, China). A total of 360 uniformly sized fish with an initial weight of 13.94 ± 0.11 g were randomly distributed into 9 cages (2.0 × 2.0 × 2.0 m), with 40 fish per cage. The cages in the pond were equipped with a disc of 100 cm diameter made of 1 mm gauze at the bottom to collect the uneaten feed. All fish were fed with a control diet (FM) for 2 weeks in order to adapt to the feeding system. After the adaptation period, the fish in three cages were fed with 40SBM, as an experimental diet, for 7 weeks, and those in another three cages were fed with 70SBM, as a positive control diet, for 7 weeks, while the left fish were still fed with FM. During the experimental period, the fish were fed to satiation four times per day for 7 weeks, and the feed rate was 2% BW/d. The experiment was conducted under a natural light and dark cycle. Water quality was tested regularly, at 26 ± 2°C, pH 7 ± 0.5, and dissolved oxygen >6.0 mg/L.

### Sampling

Thirty minutes after each feeding, the uneaten feed was collected, dried, and weighed to calculate the food intake (FI), which was measured according to the published method (Helland et al., 2017).

The fish in each cage were weighed and counted at the beginning and the end of each of the feeding trials, in order to determine the parameters for growth performance, including weight gain, % [WG(%)], specific growth rate (SGR) and feed efficiency (FE). In order to reveal the growth trend for each stage, the average daily feed intake (g/fish) was calculated for 0 days, 0 days to 3 weeks (21 days), 3 weeks (21 days) to 5 weeks (35 days), and 5 weeks (35 days) to 7 weeks (49 days). Additionally, survival rate, hepatopancreatic index (HI), intestine length index (ILI), intestosomatic index (ISI) were also calculated. Calculations were carried out using the following formulas: Weight gain, % (WG, %) = 100 × (final weight–initial weight)/initial weight; Specific growth rate (SGR) = 100 × (Final weight–Initial weight)/No. of days in trial; Feed efficiency (FE) = 100 × weight gain (g)/feed intake (g); Average daily feed intake (g/day/fish) = feed consumption / fish number/days; Survival rate = 100 × No. of live fish on day 49/No. of live fish on day 0; Hepatopancreatic index (HI) = 100 × hepatopancreas weight/body weight; Intestine length index (ILI) = 100 × intestine length/body length; Intestosomatic index (ISI) = 100 × intestine weight/body weight.

Twelve hours after feeding on 0 days, 21 days (3 weeks), 35 days (5 weeks), and 49 days (7 weeks), distal intestine (DI) and liver samples were collected from 8 to 11 fish, and immediately transferred to 4% paraformaldehyde solution for histological study [either hematoxylin-eosin (HE) staining or immunohistochemistry (IHC), 8 biological repeats per sample], or to TRIzol® Reagent (Ambion by Life Technologies) for RNA extraction (3 biological repeats per sample).

### Histological and IHC analyses

For histology, grass carp DI at different time points were analyzed by HE staining, following the previously published protocol (Lilleeng et al., 2009). The thickness of the mucosal fold (MF) was measured using ImageJ software. In order to detect hepatic lipids, which are important for hepatic pathology, oil red staining was used (Kaneko et al., 2013). The percentage of area for oil red staining dots was quantified by ImageJ software following the previously published method (Huang et al., 2017).

To detect the distribution of IgM^+^ B cells and CD4^+^ T cells, as well as the production of cytokines IL17 and IL10, IHC analysis was employed, following the previously published method (Wu et al., 2015). Commercial antibodies [anti-zebrafish CD4 monoclonal antibody (mAb), Genetex] or the antibodies developed by our group, including mouse anti-grass carp IgM (GenBank: DQ417927.1) mAb, rabbit anti-grass carp IL17 (NCBI Accession: KC978892.1) polyclonal antibody (pAb), and rabbit anti-grass carp IL10 (NCBI Accession: JQ768312.1) pAb were used in the study. Then, the mean density of fluorescent signals was measured using the ImagJ software following the previously published method (Wu et al., 2013). The calculation formula was: mean density = [∑IntDen – (Average of Mean densities for 3 background area ^*^ ∑area of signals)] / the area for the whole picture.

### RNA extraction

DI or liver tissue (100 mg) was homogenized in TRIzol® Reagent (Ambion by Life Technologies) immediately after sampling, using an electrically driven tissue homogenizer (Kimble 749540-0000) in a 1.5 ml Eppendorf tube. Then the total RNA was isolated from each sample using Tiangen RNA prep Pure animal Kit (Tiangen Biomart). All RNA samples (20 mg total RNA for each sample) were sent to Novogene Bioinformatics Technology Co. Ltd. (Beijing, China). RNA quality and quantity were determined by a Nano Photometer spectrophotometer (IMPLEN), a Qubit RNA Assay Kit in a Qubit 2.0 Flurometer (Life Technologies) and a Nano 6000 Assay Kit, which was part of the Agilent Bioanalyzer 2100 system (Agilent Technologies).

### Library preparation and sequencing for transcriptomic and mirnaomic analyses

For both the distal intestine and hepatic RNA (*n* = 3), a transcriptomic analysis was done in order to reveal their gene expression profile systematically. The procedure of the gene library preparation and sequencing for transcriptome followed previously published methods (Johnson and Hofmann, 2016). Briefly, sequencing libraries were generated using NEBNext® UltraTM RNA Library Prep Kit for Illumina® (NEB, USA) following the manufacturer's recommendations, and the library quality was assessed on the Agilent Bioanalyzer 2100 system. The library preparations were sequenced on an illumina platform and 150 bp paired-end reads were generated.

Similarly, intestinal miRNAomic analysis (*n* = 2) was done in order to reveal the timely regulation of mRNA expression by microRNA. The procedure of the library preparation and sequencing for miRNAome followed previously published methods (Gan et al., 2016). Briefly, for each distal intestine sample, 20 μg of the total RNA was separated according to size on a 15% denaturing polyacrylamide gel, and all of the 18- to 25-nt small RNA was ligated with the selected 5′ and 3′ terminus adaptors. Subsequently, the resulting 18- to 25-nt small RNA was used as a template for cDNA synthesis and was amplified by using adaptor primers for 17 cycles. The amplified products were isolated from agarose gels, and the purified DNA was sequenced with the Illumina platform and also 150 bp paired-end reads were generated.

### Gene annotation and functional analysis by GO and KEGG

All the reads in the transcriptome data were mapping to the grass carp genome (http://www.ncgr.ac.cn/grasscarp/) to get annotations. For microRNAome, Bowtie mapped the small RNA tags to reference sequences. For known miRNA, miRBase20.0 was used as the reference; modified software mirdeep2 and srna-tools-cli were used to obtain the potential miRNA. The novel miRNAs were predicted using miREvo and mirdeep2 by characterizing the hairpin structure of the miRNA precursor. Then to identify the miRNA family, miFam.dat was used for known miRNAs, and Rfam was used for the novel ones.

The revealed grass carp mRNA or miRNA was annotated by GO terms and KEGG pathways, following the previously published protocol (Xu et al., 2015; Wu et al., 2016). In brief, functional annotation and classification of genes were determined both by employing local genes blasts against Gene Ontology Consortium (http://geneontology.org/), Blast2GO (Bioinformatics Department, CIPF, Valencia, Spain), and Kyoto Encyclopedia of Genes and Genomes (KEGG) (https://www.kegg.jp/kegg/pathway.html). The enrichment of the KEGG pathways was carried out for differently expressed immune genes, including both up- and down-regulated genes during each stage, by using the gene list of grass carp immune gene library, which was described as follows.

### Construction of grass carp immune gene library

The grass carp immune gene library was constructed according to previously published methods (Xiang et al., 2010; Wu et al., 2016) with modifications based on the gene information obtained by blasting each sequence to both NCBI (ftp://ftp.ncbi.nih.gov/blast/db/FASTA/nr.gz) and Swiss-Prot (http://web.expasy.org/docs/swiss-prot_guideline.html) databases. The grass carp immune gene library contained information for immune genes at two levels (Table S1). According to both classical immunology and new advances in both fish and mammal immunology, nine categories of immune processes, including “acute phase reactions,” “pattern recognition,” “antigen processing and regulators,” “complement system,” “inflammatory cytokines and receptors,” “adapters, effectors and signal transducers,” “innate immune cells related,” “T/B cell antigen activation,” and “other genes related to immune response,” were used as the first level. Subsequently, many categories of immune genes for each immune process (detailed in the information of the library in Table S1) were applied for the second level. The library was used to filter data of transcriptome and miRNAome in order to obtain details of the immune processes as well as particular immune genes for each stage (Figure S1), during the GO and KEGG pathway enrichment, as well as the correlation analysis between transcriptome and miRNAome.

### The correlation analysis of miRNA-mRNA interaction

Target genes of miRNAs were obtained through the miRWalk database. The Pearson's correlation coefficients between miRNAs and target genes were calculated using the cor() function in R language. To obtain both the positive and negative correlations between intestinal miRNAs and target genes, the predicted target genes of miRNAs were overlapped with the identified up-regulated or down-regulated differential expressed mRNAs in each stage, respectively. All the correlations between miRNAs and target genes were subjected to construct the interaction network using Cytoscape software. Later, the genes in the grass carp immune gene library were used to select the immune-related miRNA- target gene pairs.

### RT-PCR validation

In total, 9 mRNA and 15 miRNA were tested using specially designed primers (Table S2). The same mRNA samples used for transcriptome sequencing were subjected to real-time quantitative RT-PCR (qPCR) validation (*n* = 3), following previously published protocol (Wu et al., 2016). At the same time, to validate miRNA, total RNAs were isolated using mirVana TM miRNA Isolation Kit (Ambion) according to the manufacturer's instructions and quantified using NanoDrop TM 1000 spectrophotometer (Thermo Fisher Scientific). Then miRNAs were subjected to reverse transcription by specific stem-loop RT primers supplied in TaqMan microRNA Assays kit (Applied Biosystems). The stem-loop RT-PCR was carried out following previously published method (Wang et al., 2017).

### Statistics and bioinformatics analysis

The differential expressed genes or miRNA were generated by comparing the RPKM for each gene in different time points, using the DESeq2 Rpackage (1.16.1). The resulting *P*-values were adjusted using the Benjamini and Hochberg's approach for controlling the false discovery rate (FDR). Genes or miRNA with an adjusted *P* < 0.05 found by DESeq2 were assigned as differentially expressed. In order to select immune-related transcripts using the grass carp immune gene library as well as to construct barplots for the major immune processes and immune categories, the following bioinformatics analysis was accessed. A *t*-test was used to assess differences, with FDR adjusted *p* < 0.05. Qualitative comparisons were made between samples by counting the number of differentially expressed genes. After that, the data was rearranged in EXCEL, and then was applied to plot charts by ggplot2 (2.2.1) in R language. Those immune gene categories, which were significantly (*p* < 0.05) different between 7 and 5 weeks (or 3 weeks) but not significantly different between 7 weeks and 0 days in the gut, as well as those, were significantly different between 0 days and 3 weeks (or 5 weeks, 7 weeks) in the liver, had been selected as the immune checkpoints for maintaining homeostasis. The significance of growth performance, morphological analysis and bioinformatics analysis were assessed using the parametric *t*-test run by SPSS software. The barplot was generated by Graphpad Prism software. For quantifying significance, in the growth performance analysis *p* < 0.05 was used, while in the morphological analysis *p* < 0.01 was used.

## Results

### Growth performance

**Figure 1:**
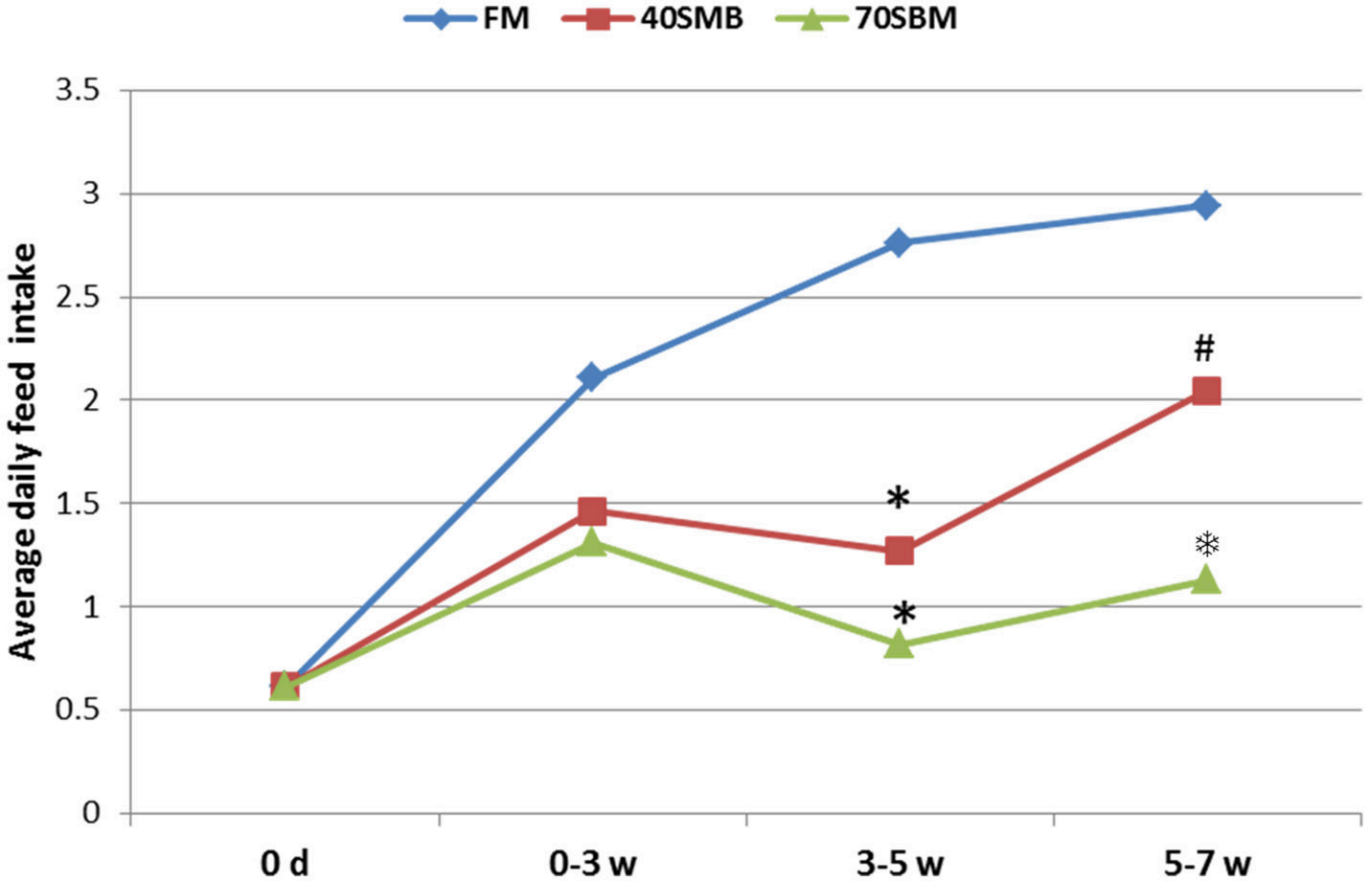
Average daily food intake (ADFI) of grass carp for different stages, including early (0–3 week), middle (3–5 week), and late (5–7 week) stages, in FM, 40SBM and 70SBM groups. The stars (^*^) represent the significant decreases while the hash (#) represents the significant increases in the food intakes compared to the previous stage for each group. The factor that ADFI in the 70SBM group was much lower than the 40SBM group was indicated by the snowflake ().

To evaluate the growth performance and survival (Table 2) of fish fed with diets containing graded levels of SBM, parameters, including initial body weight (IBW), percentage weight gain [WG(%)], feed efficiency (FE), food take (FI), survival rate (%) and specific growth rate (SGR), were measured. These parameters significantly decreased with the dietary SBM replacement. Fish in the 70SBM group showed a lower food intake (FI) (*p* = 0.001) and survival rate (*p* = 0.014) compared to other groups. However, there were no significant changes for FI and survival rate between FM and 40SBM groups. Upon the SBM replacement, the organ growth-related parameters (Table 2), including hepatopancreatic index (HI) and intestosomatic index (ISI), were significantly decreased (*p* = 0.045 for HI in 40SBM, *p* = 0.021 for HI in 70SBM, *p* = 0.016 for ISI in 40SBM, *p* = 0.008 for ISI in 70SBM), except for intestine length index (ILI). As shown in Figure 1, in 3–5 weeks (the middle stage) the average daily feed intake (ADFI) in 40SBM and 70SBM groups significantly decreased (*p* = 0.008 for 40SBM, *p* = 0.005 for 70SBM) compared to the FM group. Later, the ADFI in the 40SBM group significantly (*p* = 0.007) increased during 5–7 weeks (the late stage), yet the ADFI in the 70SBM group was significantly lower than the 40SBM group (*p* = 0.004).

**Table 2:** Growth indexes of grass carp fed with different diet for 7 weeks^1^.

	**FM**	**40%**	**70%**
IBW	13.94 ± 0.63	13.83 ± 0.58	14.06 ± 0.48
WG(%)	266.08 ± 12.24^c^	209.33 ± 15.44^b^	90.09 ± 1.02^a^
FI	59.68 ± 0.97^b^	53.73 ± 0.99^b^	34.93 ± 0.78^a^
FE	62.57 ± 1.80^c^	52.43 ± 0.97^b^	37.08 ± 1.38^a^
SGR	3.09 ± 0.08^c^	2.69 ± 0.12^b^	1.53 ± 0.01^a^
Survival rate	100.00 ± 0.00^b^	100.00 ± 0.00^b^	95.00 ± 1.67^a^
HI	3.18 ± 0.37^b^	2.41 ± 0.24^a^	2.14 ± 0.19^a^
ILI	197.09 ± 14.69	189.69 ± 12.65	202.76 ± 14.80
ISI	4.74 ± 0.44^c^	3.79 ± 0.33^b^	3.18 ± 0.31^a^

1 *Values are means ± SD, means of three replicates with sixty fish per replicate. Mean values with different superscripts in the same row are significantly different (P < 0.05). IBW: Initial body weight (g/fish), WG(%): percentage weight gain (%), FI: food intake (g/fish), FE: feed efficiency (%), SGR: specific growth rate (%/day), survival rate (%), HI: hepatopancreatic index, ILI: intestine length index, ISI: intestosomatic index. Values are means ± SD, n = 10. Mean values with different superscripts in the same row are significantly different (P < 0.05)*.

### Temporal morphological changes in the gut and liver

**Figure 2:**
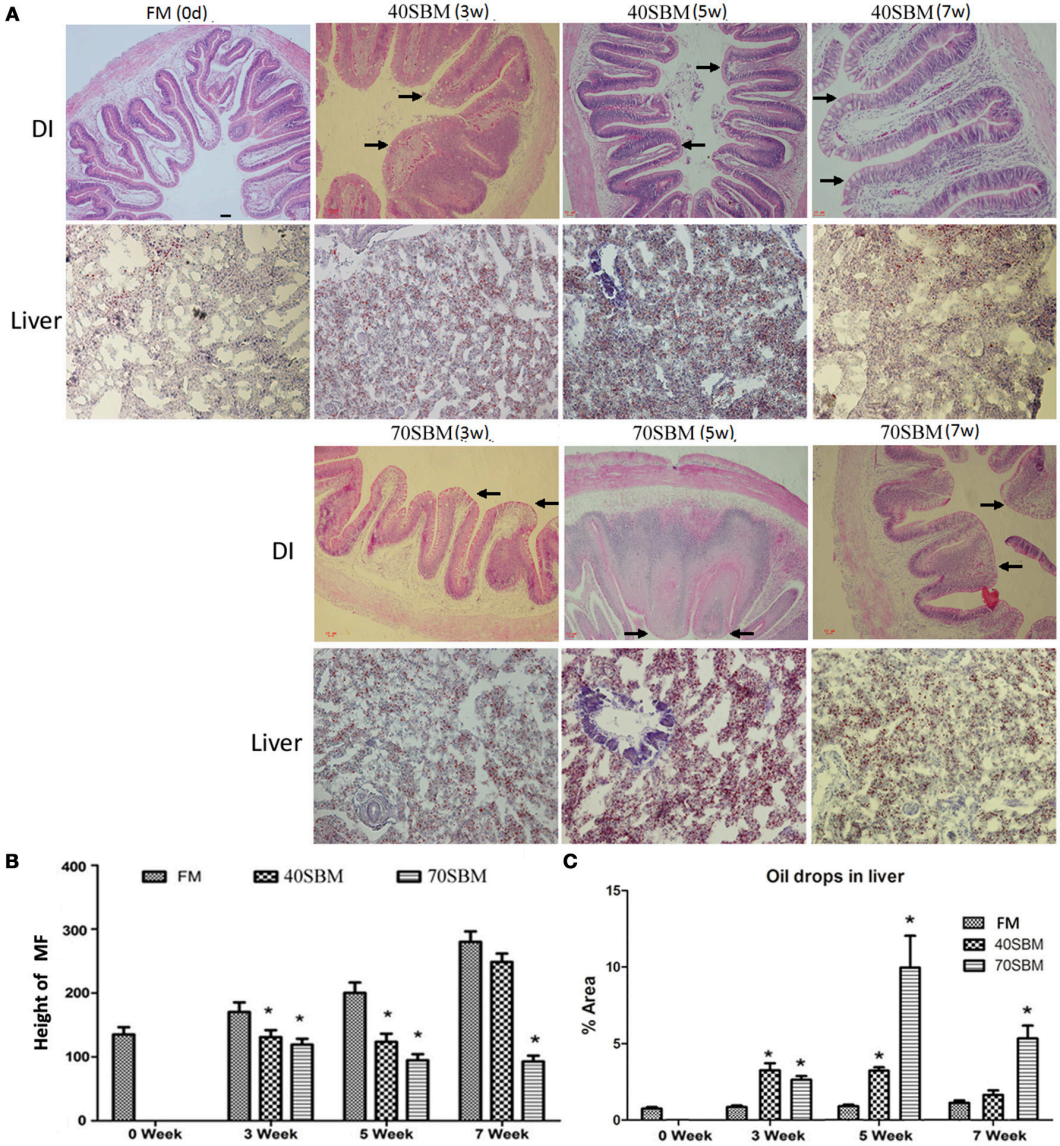
Histological analysis of the gut and liver in tested grass carp. **(A)** HE staining of DI as well as oil red staining of liver in FM, 40SBM and 70SBM groups at different time points. The typical intestinal folds with less goblet cells, widened LP, shortened MF were indicated by arrows. The chronological change for both the height of the intestinal folds **(B)** and the area of hepatic oil drops **(C)** in FM, 40SBM and 70SBM groups. The pixel was classed as an absolute length unit, and percent of area was used for quantification. Eight images per sample were used for qualification for both height of MF and the % area of oil drops. Mean ± S.D. The significant decrease for height of MF as well as significant increase for % area of hepatic oil drops was indicated by stars. The stars represent the significance of the difference between the mean of each bar for either 40SBM or 70SBM compared to FM group.

To demonstrate temporal morphological changes in the gut and liver during the feeding trials, HE staining of DI and oil-red staining of the liver were applied. No pathological alterations were observed in the FM group. After 3 weeks of SBM dietary intake at 40% inclusion, obvious morphological change for enteritis in DI was found, such as decreased volumes of goblet cells (GC), obviously widened LP and shortened MF (Figure 2A). In 40SBM group, enteritis gradually eased up at 5 weeks, and recovered at 7 weeks, as the widened LP and shortened MF became narrowed and elongated respectively gradually after 5 weeks, and even looked like normal intestinal in the FM group. In the 70SBM group, typical signs of enteritis, including shortened MF, the disappearance of the supranuclear vacuoles, increased number of goblet cells, and thickened LP and sub-epithelial mucosa, were found more obviously at 3 and 5 weeks, and then were seriously evident at 7 weeks (Figure 2A). Furthermore, the height of MF was measured (Figure 2B). The height of MF was significantly decreased at 3 and 5 weeks in both the 40SBM (*p* = 0.010 at 3 weeks, *p* = 0.006 at 5 weeks) and the 70SBM (*p* = 0.008 at 3 weeks, *p* = 0.005 at 5 weeks) groups, however at 7 weeks MF only decreased significantly in the 70SBM group (*p* = 0.002) whereas in the 40SBM group (*p* = 0.051) it recovered to a normal value. In the liver, a few oil droplets were found in the FM group, however significantly more droplets were found in both the 40SBM (*p* = 0.009 at 3 weeks, *p* = 0.000 at 5 weeks) and the 70SBM groups (*p* = 0.003 at 3 weeks, *p* = 0.016 at 5 weeks) (Figures 2A,C). In the 40SBM group oil droplets decreased after 5 weeks and then declined to a normal level at 7 weeks (*p* = 0.022), despite a thoroughly increased number of oil droplets in the 70SBM group (*p* = 0.009; Figures 2A,C).

### The temporal expression pattern of lymphocyte markers and key cytokines in gut and liver

**Figure 3:**
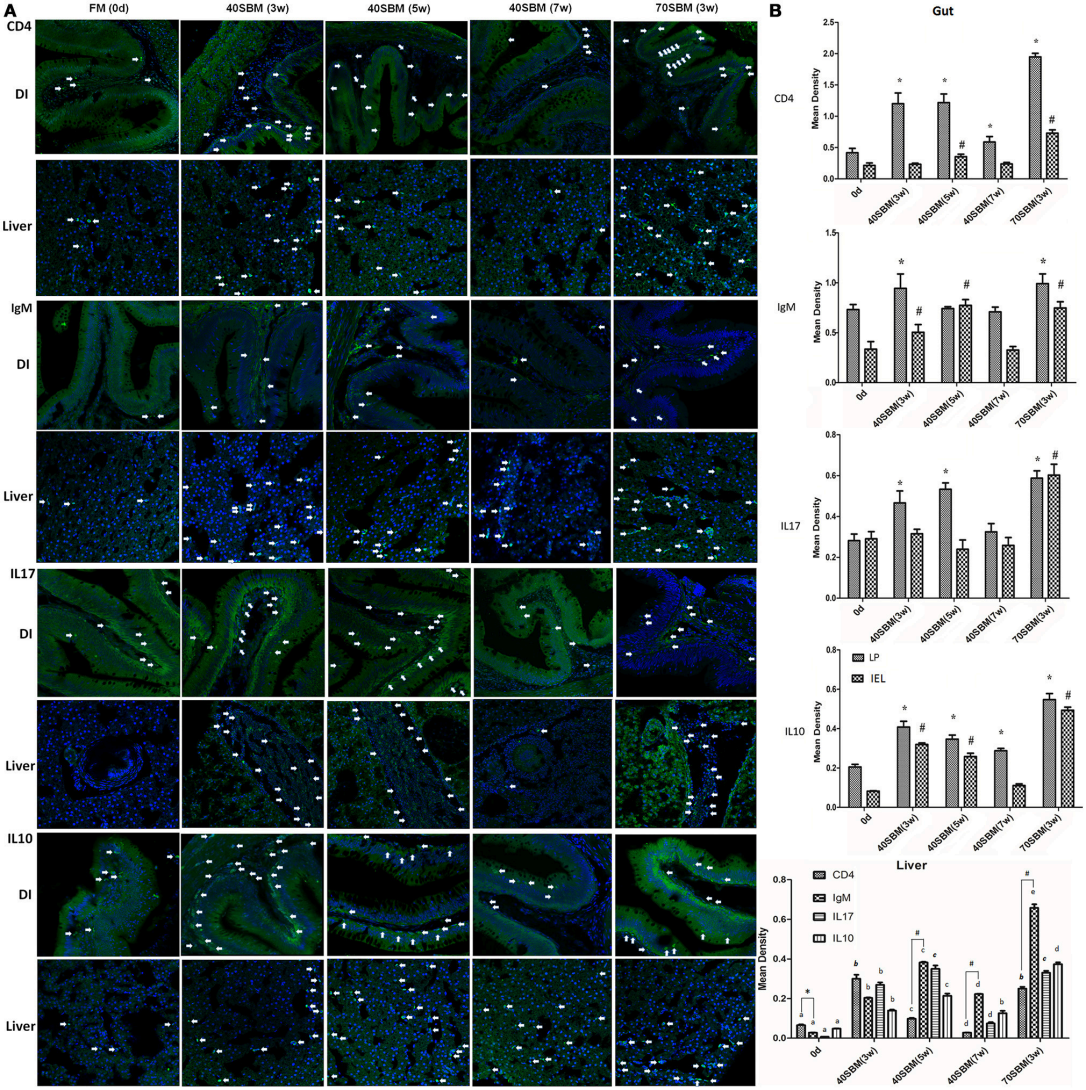
Immunohistochemistry analysis for the main lymphocytes and key cytokines involved in SBMIE. **(A)** IHC results for IgM, CD4, IL17 and IL10 in the gut and liver in all time points in 40SBM group as well as 0 days in FM group (negative control) and 3 week in 70SBM group (positive control). The green signals represent the examined protein, while the blue signals (stained by Hoechst 33342) represent the nuclei. The white arrows points out typical immunohistochemistry signals. Scale bar: 30 μm. **(B)** The quantification of IHC signals in both the gut (left) and liver (right). In the gut, the significant (*p* < 0.01) increase of signals compared to the sample at 0 days in LP or in IEL was indicated by stars or hashes. In the liver, for each protein, the signals were calculated for significant changes, indicated by lowercase letters “a, b, c, d, e”. For each protein, the same label means no difference. For comparing T cell and B cell signals, CD4 and IgM signals were calculated for *p*-value at each time point, the significant (*p* < 0.01) more CD4 was indicated by the star, whereas the significant result for IgM (*p* < 0.1) was indicated by hashes.

In addition to morphological changes, the typical signs of enteritis, lymphocytes infiltration into both LP and IEL (intestinal epithelial layer), demonstrated by IgM and CD4 IHC signals (Figure 3A) were also found in the gut. In the liver an increased IgM and CD4 signals could be found along with enteritis (Figure 3A). These typically inflammatory and anti-inflammatory related cytokines, IL17 and IL10 signals were also detected in the gut and liver (Figure 3A). All of these signals increased in the early and middle stages, and then they declined in the late stages of the 40SBM group, except for hepatic IgM and IL10 (Figure 3B). There were more intestinal signals in LP than IEL, and the IEL signals were found to have increased upon SBM exposure (Figure 3B). IgM and IL10 positive hepatic cells were still relatively higher in both number and density, compared to those in the FM group at 7 weeks (Figures 3A,B). In the 40SBM group, most signals existing in the LP at 0 days. At 3 and 5 weeks, all examined protein signals increased a lot in the LP, whereas in IEL IgM, CD4 and IL10 signals were also found to have increased. At 7 weeks only CD4 and IL10 signals still slightly higher in the LP (for CD4, *p* = 0.007; for IL10, *p* = 0.006). Regarding to the hepatic signals, CD4 highly increased earlier (at 3 weeks) than other proteins. At 5 weeks, CD4 decreased while other proteins (IgM, IL17 and IL10) increased to the maximum value. At 7 weeks, all the signals decreased. In detail, CD4 was even a slightly lower than that at 0 days (*p* = 0.003), and other proteins were still higher. IL10 kept the level similar as at 3 weeks (*p* = 0.178), and IgM still highly expressed (*p* = 0.000).

### Differentially expressed transcripts at different stages in the gut or liver by GO database analysis

At a transcriptional level, the raw, clean and mapped reads for each sample were listed in Tables S3A,B for transcriptome and miRNAome respectively. The raw data has been submitted to the Genome Sequence Archive (GSA) database (http://gsa.big.ac.cn/index.jsp) with the BioProject identifier <PRJCA000495>. In the gut, the immune-related GO terms, especially “antigen processing and presentation,” “MHC class II protein complex,” and “macrophage colony-stimulating” among up-regulated GO terms in the early stages as well as “regulation of immune effector” among down-regulated GO terms in the late stages, were revealed in Figure S2A. Likewise, in the liver the immune-related terms were only found in the early stages, including “antigen processing and presentation,” “MHC protein complex,” “MHC class II protein complex,” and “interleukin-1 receptor binding” for up-regulated GO terms, as well as “cell-cell adhesion” and “microtubule associated complex” for down-regulated ones (Figure S2B). However, for miRNA, there was no immune-related term.

### Analysis of differentially expressed transcripts at different stages in gut or liver by KEGG database

**Figure 4:**
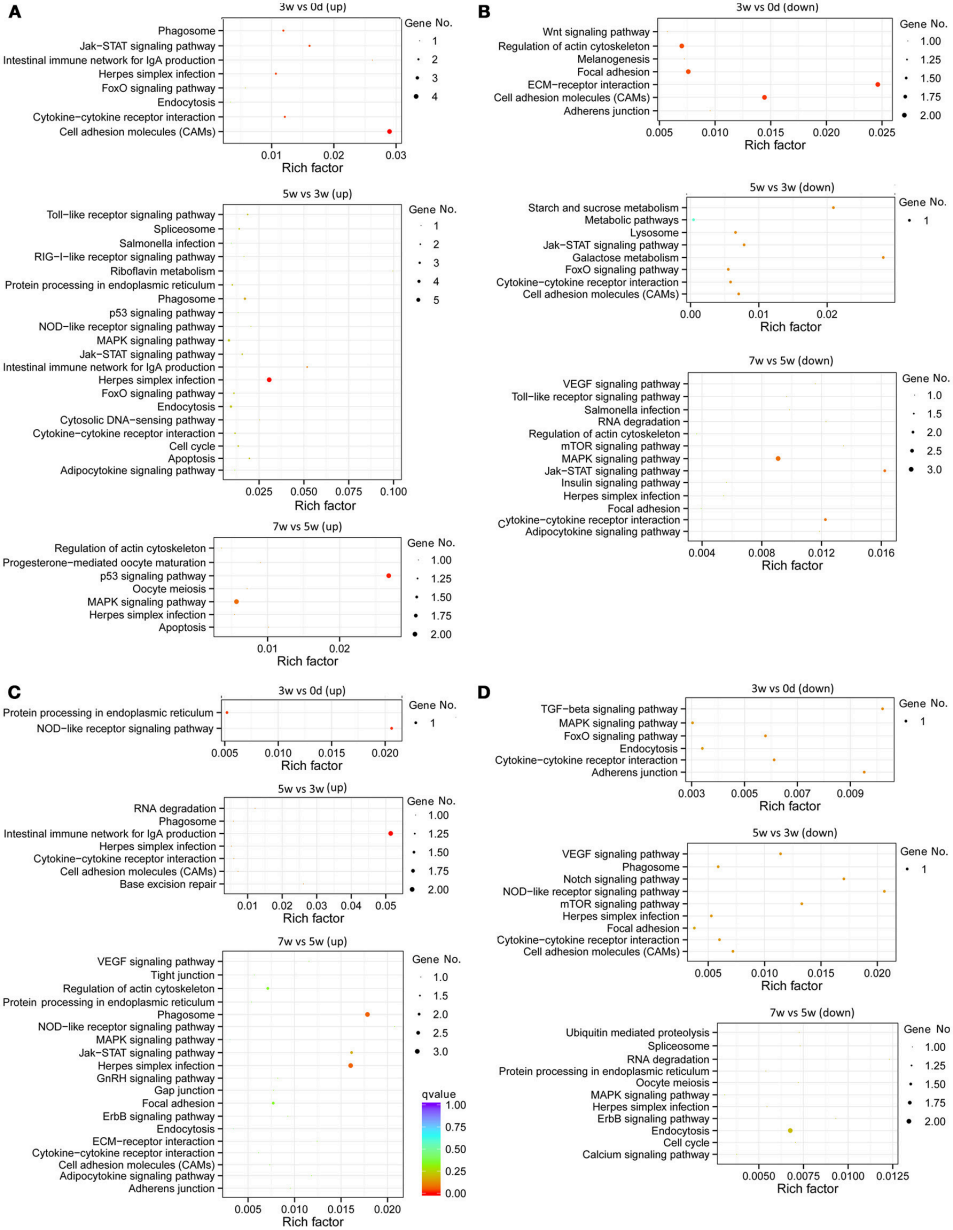
Statistics of KEGG pathway enrichment of regulated immune genes of all stages, including early (0–3 weeks), middle (3–5 weeks), and late (5–7 weeks) stages, in the grass carp gut and liver. **(A)** The pathways for intestinal up-regulated pathways. **(B)** The pathways for intestinal down-regulated pathways. **(C)** The pathways for hepatic up-regulated pathways. **(D)** The pathways for hepatic down-regulated pathways. The clusterProfiler R package was used to test the statistical enrichment of differential expression genes in KEGG pathways, and those genes in the gene list of grass carp immune gene library were selected to generate the scatter plot by the ggplot2 (2.2.1) in R package. No. of genes was indicated by the size of dots, and the rich factor was labeled as the abscissa value. *Q* < 0.05, indicated by the colors green, yellow and red, was considered significant. All dots of genes showed in this figure were included as those significant.

The immune-related KEGG pathways appeared more clearly (Figure 4), through filtering using the gene list of the grass carp immune gene library. The greatest number of intestinal immune pathways was revealed, for both up-regulated pathways in the middle stages and down-regulated pathways in the late stages (Figures 4A,B). The most enriched (rich factor > 0.02) intestinal pathways were “cell adhesion molecules” (up-regulated in the early stages), “ECM-receptor interaction” (down-regulated in the early stages), “Herpes simplex infection” (up-regulated in the middle stages), “intestinal immune network for IgA production” (up-regulated in the middle stages), “starch and sucrose metabolism” (down-regulated in the middle stages), “galactose metabolism” (down-regulated in the middle stages), and “p53 signaling pathway” (up-regulated in the late stages). Additionally, the acetylcholine in the intestinal pathway “neuroactive ligand-receptor interaction” (Figure S3) may indicate the cholinergic anti-inflammatory pathway. In the liver, the greatest number of immune-related pathways was revealed in the late stages for up-regulated pathways, while for down-regulated pathways in both the middle and late stages (Figures 4C,D). The most enriched hepatic pathways were “NOD-like receptor” (up-regulated in the early stages) and “intestinal immune network for IgA production” (up-regulated in the middle stages). Apart from the most enriched pathways, some important pathways related to inflammation developing, such as “phagosome,” “Jak-STAT signaling pathway,” and “cytokine-cytokine receptor interaction,” were also revealed in both the gut and liver.

**Figure 5:**
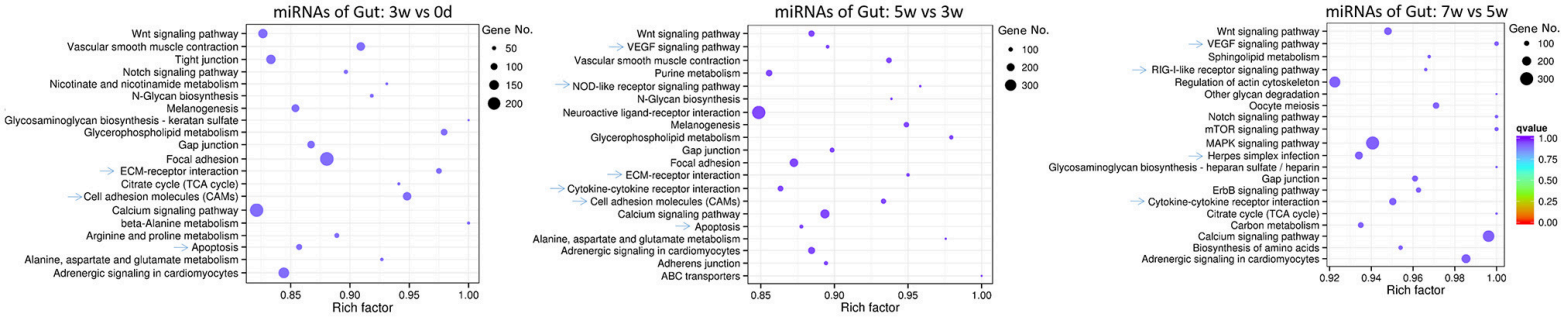
KEGG pathways for intestinal miRNAome for all stages, including early (0–3 weeks), middle (3–5 weeks), and late (5–7 weeks) stages, in grass carp. The clusterProfiler R package was used to test the statistical enrichment of differential expression miRNAs in KEGG pathways, then the ggplot2 (2.2.1) in R package was used to generate the scatter plot. No. of miRNAs was indicated by the size of dots, the rich factor was labeled as the abscissa value, and *q*-value was indicated by the color. Even though the *q*-values were not significant, the rich factors were enriched, for all the dots of miRNA in this figure.

For intestinal miRNAome, the immune-related pathways, including “ECM-receptor interaction”, “cell adhesion molecules,” and “apoptosis” were in the early and middle stages, “VEGF signaling pathway” and “cytokine-cytokine receptor interaction” were in both the middle and late stages, “NOD-like receptor signaling pathway” was only in the middle stages, with “RIG I-like receptor signaling pathway” and “herpes simplex infection” being in the late stages, were revealed (Figure 5).

### Classification of differentially expressed transcripts in gut or liver during different stages by grass carp immune gene library

**Figure 6:**
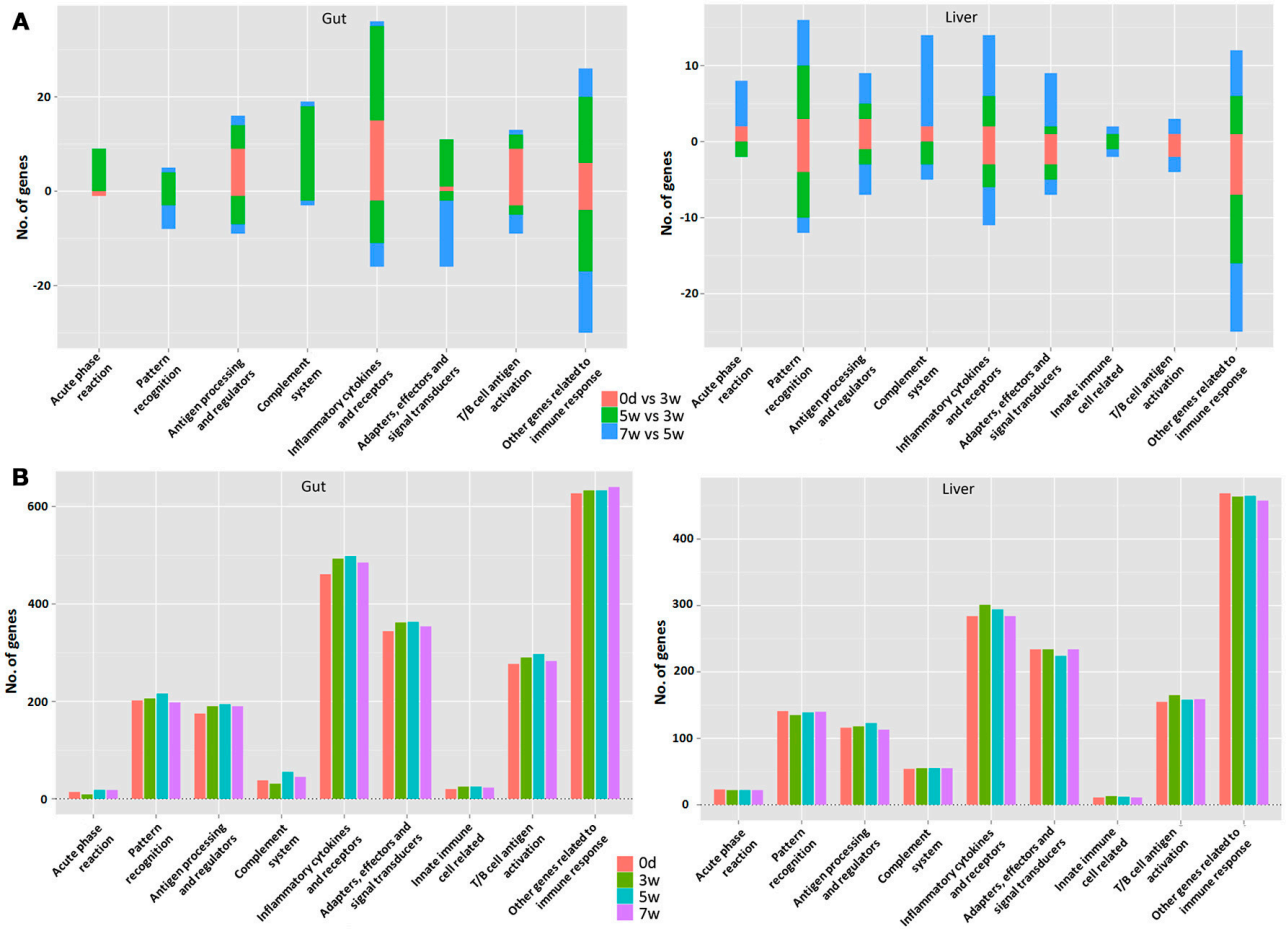
The analysis involved major immune processes in different stages in the gut (left) or liver (right) **(A)** as well as at different time points **(B)** in grass carp. “0 days vs. 3 week” refers to the early stage; “5 vs. 3 week” refers to the middle stage; “7 vs. 5 week” refers to the late stage. The bar represents the gene numbers. The up-regulated gene number is in positive value, whereas the down-regulated gene number is in negative value.

The currently constructed grass carp immune gene library (Table S1) was used to classify the revealed immune transcripts. The detailed information of differentially expressed immune genes for each stage was listed in Tables 3A,B, for intestinal and hepatic genes respectively. In the gut (Figure 6A, left) during the early stages, most immune mRNAs were up-regulated in the immune processes of “antigen processing and regulators,” “inflammatory cytokines and receptors”, and “T/B cell antigen activation”. In the middle stages, the immune mRNAs, had the largest number, containing both up- and down-regulated genes. The up-regulated genes were mainly involved in “acute phase reactions,” “complement system,” “inflammatory cytokines and receptors,” “adapters, effectors and signal transducer” etc. In the later stages, most immune mRNAs were down-regulated in the immune process such as “pattern recognition”, “inflammatory cytokines and receptors,” “adapters, effectors and signal transducers,” and “T/B cell antigen activation.” For the immune process “other genes related to immune response,” the gene numbers for up- or down-regulated pathways in either the early or middle stages were almost similar. In the later stages the immune genes were found to be mostly down-regulated. The detailed immune gene categories for intestinal immune mRNAs were listed in Table S4.

**Table 3A:** The immune processes and immune gene categories involved in differentially expressed intestinal mRNA for each stage.

**Compared group (DEG No.)**	**Immune process (Differential expressing gene No.)**	**Immune gene category**
The early stages (3 weeks vs. 0 days) UP (40)	Antigen processing and regulators (9); inflammatory cytokines and receptors (15); adapters, effectors and signal transducers (1); T/B cell antigen activation (9); other genes related to immune response (6);	**HA1**; HA2; **MHC I**; MHC I related; **MHC II; macroglobulin; CCL; CCR; chemokine receptor; FAM; FGF and related; GVIN; IFN induced proteins and relevant; IIGP; IL21R; IL34; IL7R; granzyme; CD2; CD226; CD3;** CD5; PKC; RGS; **TCR; VTCN; CD200; GIAMP; NK-lysin; ubiquitin ligase**.
The early stages (3 vs. 0 days) DOWN (11)	Acute phase reactions (1); antigen processing and regulators (1); inflammatory cytokines and receptors (2); T/B cell antigen activation (3); other gene related to immune response (4)	**Hemopexin; TGF, TGF receptor and related; integrin alpha; BCL; TCR; PTKs; CEBP;** GIAMP; lymphocyte related; **myeloid cell related**.
The middle stages (5 vs. 3 weeks) UP (83)	Acute phase reactions (9); pattern recognition (4); antigen processing and regulators (5); complement system (18); inflammatory cytokines and receptors (20); adapters, effectors and signal transducers (10); T/B cell antigen activation (3); other genes related to immune response (14).	**Fibrinogen; macroglobulin;** plasminogen; C-type lectin; **fish-egg lectin; galectin; intelectin; CDK and related; C1q; C3; C5; C7; C8; C9**; CFB; CFI; **MASP**; CCR; **CFLAR; CXCR**; EGF, EGFR and related; **GVIN**; IFN induced proteins and relevant; **IRF**; NFIL3; CD276; CIS; lysosomal protein; **NFKBI and related; NLRC3**; SCOCS (1-7); BCL; **FCGBP; RGS; caspase; CEBP**; hepcidin; HSP; myeloid cell related; **oncogene; ubiquitin ligase**.
The middle stages (5 vs. 3 weeks) DOWN (37)	Pattern recognition (3); antigen processing and regulators (6); complement system (2); inflammatory cytokines and receptors (9); adapters, effectors and signal transducers (2); T/B cell antigen activation (2); other genes related to immune response (13).	**LPS-anchor protein; LRR-containing proteins; CDK and related**; MAF; **MHC I related; TNF; TNR and related; C3aR; MASP; FAM; GVIN; IL34**; IL4; **IL7R**; lysosomal protein; **NLRC3; CD2; PIGR; GIAMP; HSP**; ubiquitin ligase.
The late stages (7 vs. 5) UP (12)	Pattern recognition (1); antigen processing and regulators (2); complement system (1); inflammatory cytokines and receptors (1); T/B cell antigen activation (1); other genes related to immune response (6).	**HMG; CDC and related; MHC I related; C6; CCR; CD48; caspase; CD82; GIAMP; ubiquitin ligase**.
The late stages (7 vs. 5 weeks) DOWN (44)	Pattern recognition (5); antigen processing and regulators (2); complement system (1); inflammatory cytokines and receptors (5); adapters, effectors and signal transducers (14); T/B cell antigen activation (4); other genes related to immune response (13).	**LPS-anchor protein; LRR-containing proteins**; CDK and related; **VEGF; C1q; CXCL; FGF and related; GVIN; IL20R; CD276; CIS**; lysosomal protein; **MAPK; MAPK related; NLRC3; PPARA**; SOCS (1-7); **BCL; NFAT**; SEMA; caspase; **GIAMP; HSP; oncogene; ubiquitin ligase; ubiquitin related**.

*The bold immune gene categories were found regulate by miRNA, as shown in Table 5 and Tables S8–S10*.

**Table 3B:** The immune processes and immune gene categories involved in differentially expressed hepatic mRNA for each stage.

**Compared group (DEG No.)**	**Immune process (differential expressing gene No.)**	**Immune gene category**
The early stages (3 vs. 0 days) UP (15)	Acute phase reactions (2); pattern recognition (3); antigen processing and regulators (3); complement system (2); inflammatory cytokines and receptors (2); adapters, effectors and signal transducers (1); T/B cell antigen activation (1); other genes related to immune response (1).	Macroglobulin; c-type lectin; LRR-containing proteins; nattectin; LRMP; MHC II; C1q; C1q and TNF related protein; chemokine receptor; GVIN; perforin 1; Ig light chain; myeloid cell related.
The early stages (3 vs. 0 days) DOWN (20)	Pattern recognition (4); antigen processing and regulators (1); inflammatory cytokines and receptors (3); adapter, effectors and signal transducers (3); T/B cell antigen activation (2) other genes related to immune response (7).	LRR-containing proteins; TGF, TGF receptor and related; FAM; TTN/TITIN; MAPK related; NFKBI and related; PPARA; SEMA; CEBP; DMBT; microtubule-associated; ubiquitin ligase; ubiquitin related.
The middle stages (5 vs. 3 weeks) UP (20)	Pattern recognition (7); antigen processing and regulators (2); inflammatory cytokines and receptors (4); adapters, effectors and signal transducers (1); innate immune cells related (1); other genes related to immune response (5).	HMG; LRR-containing proteins; scavenger receptor; CDK and related; HA2; CCR; FAM; integrin beta; CD276; MMD; CDBP; HSP; ubiquitin ligase.
The middle stages (5 vs. 3 weeks) DOWN (28)	Acute phase reactions (2); pattern recognition (6); antigen processing and regulators (2); complement system (3); inflammatory cytokines and receptors (3); adapters, effectors and signal transducers (2); innate immune cells related (1); other genes related to immune response (9).	Fibrinogen; macroglobulin; LRR-containing proteins; nattectin; NOD; LRMP; VEGF; C1q; C3; CFB; FAM; ILDR; TTN/TITIN; programmed cell death protein; DEDD; NCAM; GIAMP; HSP; platelet related; ubiquitin ligase.
The late stages (7 vs. 5 weeks) UP (52)	Acute phase reactions (6); pattern recognition (6);antigen processing and regulators (4); complement system (12); inflammatory cytokines and receptors (8); adapters, effectors and signal transducers (7); innate immune cells related (1); T/B cell antigen activation (2); other genes related to immune response (6).	Macroglobulin; fibrinogen; c-type lectin; HMG; LRR-containing proteins; CDK and related; LRMP; C1q; C3; C4; CFB; CFH; CFI; CCL; CRELD; FAM; ILDR; integrin alpha; LIFR; NFIL3; CIS; MAPK related; NFKBI and related; NLRC3; PPARA; RIP; TRAF related; MMD; GIAMP; HSP; myeloid cell related; oncogene; ubiquitin ligase; ubiquitin related.
The late stages (7 vs. 5 weeks) DOWN (27)	Pattern recognition (2);antigen processing and regulators (4); complement system (2); inflammatory cytokines and receptors (5); adapters, effectors and signal transducers (2); innate immune cells related (1); T/B cell antigen activation; (2).other genes related to immune response (9).	Galectin; CDC; CDK and related; C1q; C7; ACRs; EGF, EGFR and related; FAM; GVIN; programmed cell death protein; CD276; MMD; MAL; RGS; CEBP; GIAMP; HELLS; HSP; ubiquitin ligase; ubiquitin related.

Among hepatic mRNAs (Figure 6A, right), the immune gene numbers increased gradually from the early to late stages and the largest quantity was found in the late stages. In the early stages, most immune genes were down-regulated, especially in the immune process such as “pattern recognition,” “inflammatory cytokines and receptors,” “adapters, effectors and signal transducers,” and “other genes related to immune response.” A small amount of up-regulated immune genes were mainly involved in “acute phase reactions,” “antigen processing and regulators,” and “complement systems.” In the middle stages, the gene numbers for up- or down-regulated were almost similar. In the late stages, the up-regulated mRNAs increased greatly, especially in the immune processes “acute phase reaction,” “pattern recognition,” “complement system,” “inflammatory cytokines and receptors,” and “adapters, effectors and signal transduction.” Nevertheless, for the immune process of “other genes related to immune response,” the genes were mainly down-regulated, especially in the early stages. The detailed immune gene categories for hepatic immune mRNAs were listed in Table S5.

### Classification of time point specific transcripts in gut or liver by grass carp immune gene library

Generally speaking, more immune transcripts were revealed in the gut compared to the liver, except for those involved in “acute phase reactions” and “complement systems.” The intestinal immune gene number increased obviously from 0 days to 5 weeks, and then at 7 weeks decreased in most immune processes (Figure 6B, left). Yet, in the liver (Figure 6B, right), the increased immune gene number from 0 days to 5 weeks was not as obvious as that in the gut, and even with decline in many immune processes, and the decreased immune gene number at 7 weeks was seldom observed, only in “antigen processing and regulators” and “inflammatory cytokines and receptors.” The details for time point-specific transcripts were listed in Tables S6, S7 for the gut and liver respectively. The intestinal or hepatic immune checkpoint genes (Table 4) responsible for maintaining homeostasis were detailed for the involved immune processes and immune categories. Later, qPCR validation result of 23 reactions of 15 genes that were randomly selected demonstrated that fold-changes between transcriptome and qPCR results correlated well (Table S2).

**Table 4:** The immune processes, immune gene categories and gene annotation for the immune checkpoints of homeostasis in the gut or liver.

**Immune process**	**Immune gene category and gene annotation in gut**	**Immune gene category and gene annotation in liver**
Acute phase reactions	**Plasminogen:** plasminogen, plasminogen-like.	-
Antigen processing and regulators	**MHC I related:** major histocompatibility complex class I-related gene, Class I histocompatibility antigen.	-
Inflammatory cytokines and receptors	**IFN induced proteins and relevant:** gamma-interferon-inducible lysosomal thiol reductase, interferon-induced 35 kDa protein, interferon-induced guanylate-binding protein 1, interferon-induced guanylate-binding protein 2 interferon-induced helicase C domain-containing protein 1, interferon-induced protein with tetratricopeptide repeats 1, interferon-induced protein with tetratricopeptide repeats 1B, interferon-induced protein with tetratricopeptide repeats 5, interferon-induced transmembrane protein 3, interferon-induced very large GTPase 1, interferon-induced, double-stranded RNA-activated protein kinase, interferon-inducible 58 kDa protein, interferon-inducible double stranded RNA dependent activator, interferon-inducible double-stranded RNA-dependent protein kinase, interferon-inducible protein IFI56, interferon-related developmental regulator 1, interferon-related developmental regulator 2, interferon-stimulated 20 kDa exonuclease-like 2.	**IFI:** interferon-induced protein 44, interferon-induced protein 44-like; **IIGP:** interferon-inducible GTPase 5.
T/B cell antigen activation	**BCR:** B-cell receptor CD22-like, B-cell receptor CD22.	**JAG:** jagged-1a, jagged-1b, jagged-2-like; **Ig light chain:** Ig kappa chain C region, Ig lambda-3 chain C region, immunoglobulin lambda-like polypeptide 1, Ig kappa chain V-I region Bi; **LAG:** lymphocyte activation gene 3 protein, lymphocyte activation gene 3 protein-like.
Other genes related to immune response	**Leukocyte related:** leukocyte elastase inhibitor, leukocyte elastase inhibitor A, leukocyte receptor cluster member 8, leukocyte receptor cluster member 9, leukocyte receptor cluster member 1, leukocyte surface antigen CD53.	-

*The bold values denotes Immune gene category*.

### The correlation analysis of intestinal immune related MIRNA and MRNA

**Figure 7:**
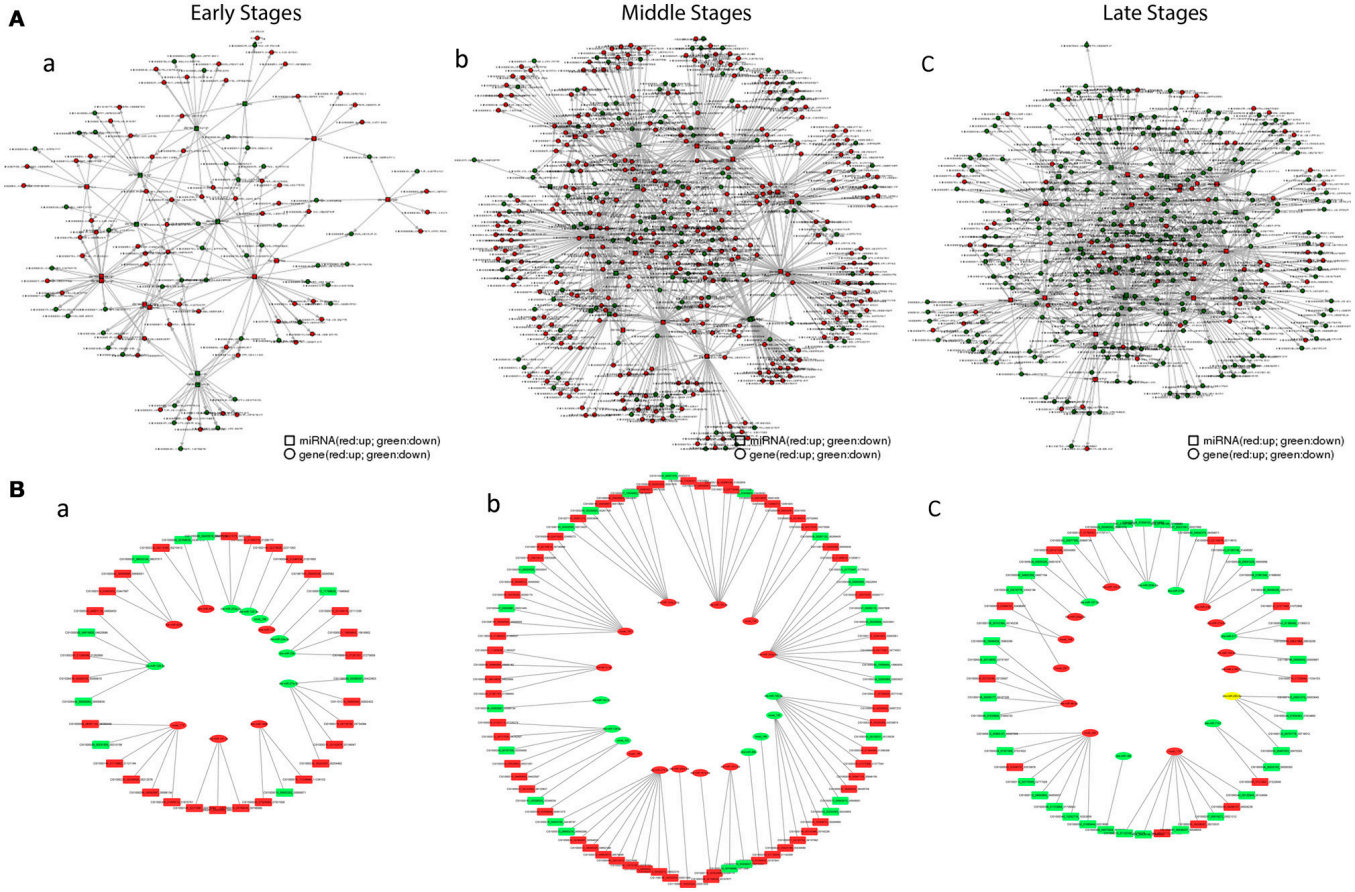
The interaction network of miRNAs combines with mRNAs. **(A)** The relationship between total mRNAs and related miRNAs. Red circles and boxes represent up-regulated mRNA and miRNA respectively, whiles green circles and boxes represent down-regulated mRNA and miRNA. **(B)** The relationship between immune mRNAs and related miRNA. Red boxes and ovals represent up-regulated mRNAs and miRNAs respectively, whiles green ones represent down-regulated ones. (a) in the early stages; (b) in the middle stages; (c) in the late stages.

For the correlation analysis between mRNA and miRNA (Figure 7A), during the early stages, the miRNA regulation on mRNA was minor, whereas the regulation was found mostly in the middle stages and then regulation heavily declined in the late stages. Furthermore, similar trends were also revealed after filtered by the grass carp immune gene library (Figure 7B). The matched pairs for miRNA and mRNA were not only positive but also had a negative correlation. The intestinal immune-related pairs of miRNAs and their target mRNAs in each stage were listed in Table 5, and the details were in Tables S8–S10 for each stage. In the stem-loop qPCR validation result, among 16 reactions of randomly selected 13 miRNAs, fold-changes between miRNAome and qPCR results correlated well (Table S2).

**Table 5:** The immune gene related miRNAs and their target mRNAs in each stage in the gut.

**Stage**	**Immune gene related miRNAs and their targets**	**The immune progress for target mRNA (Gene NO.)**	**The immune gene category of target mRNA**
From day 0 to 3 weeks (the early stages)	**Up:** dre-miR-462 (hemopexin); novel_179 (TGFB1); dre-miR-146b (PTK6); dre-miR-429b (MCL-1).	**Down:** acute phase reactions (1); antigen processing and regulators (1); T/B cell antigen activation (1); other genes related to immune response (1).	Hemopexin; TGF, TGF receptor and related; PTKs; Myeloid cell related.
	**Up:** dre-miR-462 (CML1); dre-miR-429b (CCL8, TRIM21); novel_179 (IL7Rα, NEURL3, CD2, CD3, GIMA8); dre-miR-141-3p (HA2D, GIMA7); dre-miR-146b (Granzyme K, CCR4); dre-miR-731 (IFIT1).	**Up:** antigen processing and regulators (2); inflammatory cytokines and receptors (5); adapters, effectors and signal transducers (1); T/B cell antigen activation (2); other genes related to immune response (4).	HA1; MHC II; CCL; CCR; chemokine receptor; IFN induced proteins and relevant; IL7R; granzyme; CD2; CD3; GIAMP; ubiquitin ligase.
	**Down:** dre-miR-129-5p (CEBPD, TRBC2); dre-miR-27a-3p (ITA9); dre-miR-23a-3p (BC11A); dre-miR-203a-3p (ITA9).	**Down:** inflammatory cytokines and receptors (2); T/B cell antigen activation (2); other genes related to immune response (1).	Integrin alpha; TCR; BCL; CEBP.
	**Down:** dre-miR-129-5p (B2MG, IIGP5, TCB1); dre-miR-132-3p (HB23); dre-miR-203a-3p (CD226, CD200); dre-miR-23b (VTCN, FA46A, FGFR1); dre-miR-27a-3p (CD3E, NK-lysin, HMR1, GVIN1); novel_188 (IL21R).	**Up:** antigen processing and regulators (3); inflammatory cytokines and receptors (5); T/B cell antigen activation (4); other genes related to immune response (2).	Microglobulin; MHC II; MHC I; IIGP; FAM; IL21R; FGF and related; GVIN; TCR; CD226; VTCN; CD3; CD200; NK-lysin.
From 3 to 5 weeks (the middle stages)	**Up:** dre-miR-203a-3p (HSF2, TP8L1, GIMA4, GVIN1, CDKL5, ADNP2); novel_748 (IL7RA); dre-miR-725-3p (MASP1, NLRC3); dre-miR-133a-2-5p (FA46A, GIMA7, HMR1,); novel_795 (GIMA7, GVIN1); dre-miR-27b-3p (F173B, FLRT3); dre-miR-181c-5p (PIGR, GIMA8).	**Down:** pattern recognition (1); antigen processing and regulators (3); complement system (1); inflammatory cytokines and receptors (5); adapters, effectors and signal transducers (1); T/B cell antigen activation (1); other genes related to immune response (6).	LRR-containing proteins; MHC I related; CDK and related; TNF, TNR and related; MASP;FAM; IL7R;GVIN; NLRC3; PIGR; ADNP; GIAMP;HSP.
	**Up:** dre-miR-203a-3p (FAM84B, IFT1B, NFIL3, GVIN1, C1QL4); novel_748 (PLMN, HSP71); dre-miR-725-3p (CCR9, MUG2, SOCS3, CLC10, GVIN1, RNF182); dre-miR-133a-2-5p (CDK8, HEPC1, ES8L3, NLRC3, GVIN1, I2B2A); novel_795 (C8β, CASP3, A2MG, FEL, NLRC3); dre-let-7c-3p (MASP2, RGS3, CEBPD, FIBA); novel_186 (galectin-9); dre-miR-27b-3p (FCGBP, A2MG, C5, FIBG, C9, GVIN1); dre-miR-200a-5p (FAM173A); dre-miR-181c-5p (intelection).	**Up:** acute phase reactions (6); pattern recognition (4); antigen processing and regulators (1); complement system (5); inflammatory cytokines and receptors (12); adapters, effectors and signal transducers (3); T/B cell antigen activation (2); other genes related to immune response (5).	Fibrinogen; macroglobulin; plasminogen; C-type lectin; fish-egg lectin; galectin; intelectin; CDK and related; C1q; C5; C8; C9; MASP; CCR; EGF, EGFR and related; FAM; GVIN; IFN induced proteins and relevant; IRF; NFIL3; NLRC3; SOCS (1-7); FCGBP; RGS; caspase; CEBP; hepcidin; HSP; ubiquitin ligase.
	**Down:** novel_158 (C3AR); dre-miR-10b-5p (IL34, LITAF); dre-miR-129-5p (GIMA7); novel_103 (TNF10); dre-miR-10c-3p (CD2).	**Down:** pattern recognition (1); antigen processing and regulators (1); complement system (1); inflammatory cytokines and receptors (1); T/B cell antigen activation (1); other genes related to immune response (1).	LPS-anchor protein; TNF, TNR and related; C3aR; IL34; CD2; GIAMP.
	**Down:** novel_158 (c-FOS, IKBA, CASP7, RN19A); dre-miR-10b-5p (C3, CXCR4, FIBB, FIBA, CDK13, GVIN1); dre-miR-129-5P (C7, C1q, IRF), novel_103 (C1RA); dre-miR-26b (TRIM21); novel_146 (CFLAR).	**Up:** acute phase reactions (2); antigen processing and regulators (1); complement system (4); inflammatory cytokines and receptors (4); adapters, effectors and signal transducers (1); other genes related to immune response (4).	Fibrinogen; CDK and related;C3; C1q; C1r; C7; CFLAR; CXCR;GVIN; IRF; NFKBI and related; caspase; oncogene; ubiquitin ligase.
From 5 to 7 weeks the late stages)	**Up:** novel_748 (CASP7);dre-miR-96-5p (PRGC2, M3K2, M3K13, LYST);novel_280 (CD276, BC11A, SOCS3, RNF213, CISH, HECTD4);novel_179 (CD276, c-FOS, UBP2, GIMA1, C1QL4);dre-miR-100-5p (GVIN1);dre-miR-27a-5p (M3K11);dre-miR-23b (HSP60, PRGC1);dre-miR-182-5p (UBP34);dre-miR-200a-5p (NLRC3);novel_297 (FGF10).	**Down:** complement system (1); inflammatory cytokines and receptors (2); adapters, effectors and signal transducers (11); T/B cell antigen activation (1); other genes related to immune response (9).	C1q; GVIN; FGF and related; CIS; MAPK;MAPK related; PPARA; NLRC3; Lysosomal protein; CD276; SOCS (1-7); BCL;GIAMP; HSP; oncogene; caspase; ubiquitin ligase.
	**Up:** novel_748 (HA1F); dre-miR-96-5p (CD82), novel_280 (GIMA8), novel_179 (CASP3, GIMA8, CD48), dre-miR-2188-3p (CCR4), dre-miR-182-5p (HBP1).	**Up:** pattern recognition (1); antigen processing and regulators (1); inflammatory cytokines and receptors (1); T/B cell antigen activation (1); other genes related to immune response (3).	HMG; MHC I related; CCR; CD48; CD82; GIAMP; caspase.
	**Down:** dre-miR-184 (LRBA), dre-miR-7147 (CXL10), dre-miR-205-5p (RN213, IL20Rα, RNF182, NLRC3), dre-miR-217 (PRGC1), dre-miR-216a (LRWD1, NFAT5), dre-miR-203b-3p (VGFAA, BC11A, LRRK1, GVIN1).	**Down:** pattern recognition (3); antigen processing and regulators (1); inflammatory cytokines and receptors (3); adapters, effectors and signal transducers (2); T/B cell antigen activation (2); other genes related to immune response (2).	LPS-anchor protein; LRR-containing proteins; VEGF; CXCL; GVIN; IL20R; NLRC3; PPARA; BCL; NFAT; ubiquitin ligase.
	**Down:** dre-miR-7147 (C6), dre-miR-217 (RN152), dre-miR-137-3p (GIMA7).	**Up:** antigen processing and regulators (1); complement system (1); other genes related to immune response (2).	CDK and related; C6; GIAMP; ubiquitin ligase.

## Discussion

In order to reveal the self-recovery mechanism of fish SBMIE, both histological and integrative transcriptional analysis of the gut and liver for immune status in a typical herbivorous fish was conducted. The current studies result showed that the resilience to SBM stress does exist in grass carp. At a histological level, the morphology of intestinal folds recovered, and the inflammative oil droplets (Li et al., 2009) in the liver decreased at 7 weeks. Regarding the protein levels, the relatively higher expression of hepatic IgM and IL10, as well as many IEL IL10 at 7 weeks in the 40SBM group suggested a tolerance. Transcriptomic analysis reflected that both the gut and liver in the grass carp had acute inflammation, which then eased up, though using different strategies.

It is worth mentioning that the current study revealed that immune checkpoints provide key factors in fish gut and liver, for maintaining immune homeostasis upon foodborne enteritis. Among intestinal immune checkpoint genes, the IFN induced proteins have been reported with protective roles in mucosal immunity (Lai et al., 2008; Hsu et al., 2013). Intestinal plasminogen, which could interact with probiotics (Wei et al., 2014), suggested the homeostasis state. MHC I and related genes suggested that MHC-restricted self-recognition may suppress Th1 response (Das et al., 2003). CD22, as a regulatory factor preventing over-activation of the immune system (Hatta et al., 1999), could be a marker for homeostasis. In addition, the leukocyte protease inhibitor has been proposed as a new therapeutic target for enteritis (Vergnolle, 2016). Among hepatic immune checkpoint genes, less Ig light chain deposition at a healthy state may help protection from tissue damage (Brilland et al., 2016). LAG-3, which could negatively regulate T cell proliferation, activation and homeostasis (He et al., 2016) may be a key regulator for liver immunity.

In regard to enteritis related immune genes, many clues indicating the resilience to SBM stress were revealed. The up-regulated CD2 and CD3 indicated T cell proliferation at the beginning. IL17 transcript was found in both the gut and liver only in the middle stages, even though its protein could be detected earlier. The typical T cell activation signal CD276, up-regulated in middle stages and down-regulated in the late stages for both the gut and liver, indicating the turnover from inflammation to remission. Moreover, since IL7 promotes naive intestinal T-cell homing (Kerdiles et al., 2009; Cimbro et al., 2012) and IL7R indicates T cell development (Palmer et al., 2008), IL7R, up-regulated in the early stages but down-regulated in the middle stages, which indicated the diminishing of enteritis. The up-regulated NF-κB inhibitor and SOCS3, which could inhibit JAK1, JAK2 or TYK2 signaling (Babon et al., 2012), in the middle stages, this also suggested the trend for remising. Additionally, down-regulation of CISH (Cytokine-inducible SRC homology 2 domain protein), indicated de-suppression of Treg (Trengove and Ward, 2013; Liu et al., 2015) and attenuation of CTL (Miah and Bae, 2013) in the late stages. In the liver, both the NF-κB inhibitor and PPARα, down-regulated in the early stages and up-regulated in the late stages, indicating the final relief of hepatic inflammation and lipid metabolism disorder (Tyagi et al., 2011). On the other hand, the adjustment of feed intake also contributed to resilience. Intestinally absorbed antinutritional factors and their metabolites from soybean meal negatively affected fish performance. However, the factor of drop-off of the average daily feed intake during the middle stages led to less stimulation of antinutritional factors (Zhou et al., 2018), for which provisions were made for remising in the later stages.

For the factors involved in enteritis processing, the immune-related pathways demonstrated intestinal inflammation in the early and middle stages. The enhanced gene expression in the pathway “phagosome” suggested the involvement of a macrophage, as the main intestinal phagocyte during enteritis (Grainger et al., 2017). The “Jak-STAT signaling pathway,” up-regulated during the early stages but down-regulated in the middle stages, suggested that acute inflammation was finished. Interestingly, the fact that the pathway “neuroactive ligand-receptor interaction” was always found to be active for acetylcholine related genes, indicated the possibility of an existing intestinal cholinergic anti-inflammatory pathway (Bonaz et al., 2018).

In the early stages, the T cell related transcripts were the most apparent. Because many unconventional CD8^+^ NKT cells in DI (Bannai et al., 2001) together with NK-lysin, as an effector of cytotoxic T and NK cells in GALT (Andersson et al., 1995; Hirono et al., 2007; Chen et al., 2015), the up-regulation of CD2, the marker for T and NK cells, as well as NK-lysin, suggested a CTL response upon the onset of SBMIE. The up-regulated IL21R also suggested B cell maturation, since the B cell differentiation required direct IL21R signals (King et al., 2010). In addition, the down-regulated MCL1 mediated by miR-429b suggested that there was a weakening in MCL1's protection from epithelial apoptosis (Lv et al., 2014; Nijhuis et al., 2017). This was in accordance with the finding that SBM diet increased turnover of intestinal epithelial cells (Chikwati et al., 2013). In the middle stages, innate factors played a vital role. The up-regulated galectin 9 might suppress allergies, suppress activation, restore phagocytic capacity, and normalize CD103 expression in intestinal DCs (de Kivit et al., 2017). The up-regulated GVIN (interferon-induced very large GTPase) indicated an enhancing of cell-autonomous immunity, because of its central role in host defense (Kim et al., 2012). Both up-regulated complement components and down-regulated MASP (mannose-binding lectin-associated serine protease), demonstrated an activating of complement cascades. Additionally, up-regulated acute phase proteins could also activate the complement system (Jain et al., 2011). Beyond innate factors, down-regulated pIgR suggested decreased sIgT, for that pIgR could transport tetrameric IgT across mucosal epithelia (Zhang et al., 2010).

Although enteritis occurred, many signs of recovery appeared in the late stages. Down-regulation of the inflammasome component NLRC3 (Kim et al., 2015) suggested an end to inflammation. Down-regulated IL20Rα, involved in signaling for wound-healing cytokine IL20 (Kolumam et al., 2017), indicated intestinal recovery. This is also in line with findings from patients who recovered from ulcerative colitis (Fonseca-Camarillo et al., 2013). Recently, a modulatory role of CD48, for creating intracellular bacterial reservoirs in mast cells (St John and Abraham, 2013), and the antigen tolerance of T cell (McArdel et al., 2016) has been suggested. Therefore, the up-regulated CD48 may indicate intestinal tolerance. Also, the up-regulated CD82, as a component of intestinal epithelial exosomes, indicate the recovery of IEL antigen presenting (Mallegol et al., 2007).

Despite the main contribution of mRNAs, miRNAs also play a vital role during immune processes in SBMIE. The miRNA related KEGG pathways refer to pattern-recognition receptors (PRRs) existed in both the middle and late stages. The “NOD-like receptor signaling pathway” in the middle stages was coincident with enteritis related miRNA regulation for NOD2 signaling pathway (Lipinski et al., 2012; Warner et al., 2014). The “RIG-I-like receptor signaling pathway” in the late stages was in line with the recent finding that RIG-I/MAVS and STING signaling could promote gut integrity (Fischer et al., 2017). For further detail about miRNAs, both mammalian enteritis related and novel miRNAs have been found. The current miRNA results contain the IBD related miRNAs, such as miR-10, which could down-regulate mucosal inflammatory response in DC, and miR-132, which was revealed to enhance the cholinergic anti-inflammatory pathway via regulation on AchE (acetylcholinesterase) (Cao et al., 2017). This also predicted the inhibiting role of novel 280 and novel 179 on CD276, which may suggest their therapeutic potential. In addition, the miRNA-mRNA's negative correlation indicated that miRNA-related activating (Portnoy et al., 2011; Jiao and Slack, 2014) also exist in fish.

Along with the recovery of enteritis, rebalancing of hepatic immune responses also suggests the ongoing remising after acute inflammation, although with later and longer reactions. In the early stages, the up-regulated C1q indicated enhancing of phagocytosis, and the up-regulated nattectin indicated Th1 response (Lv et al., 2016). The increased Ig light chain may suggest tissue damage (Baden et al., 2009). In the middle stages, the pathways “intestinal immune network for IgA production,” and “cytokine-cytokine receptor interaction” indicated acute inflammation. The down-regulated VEGF-A, was responsive to recruits macrophages (Walczak et al., 2004; Tanaka and Iwakiri, 2016), indicating the finishing of the acute immune response. Whereas, the up-regulated CCR9, a marker of gut-homing lymphocyte, suggested the entering of intestinal activated lymphocytes (Eickmeier et al., 2014). This may relate to hepatic inflammation, since the gut-primed T cells, preferentially migrating to the liver, could induce autoimmune disease (Eickmeier et al., 2014). The up-regulated scavenger receptor cysteine-rich type I protein M130, indicated macrophage's alternative-activation (Polfliet et al., 2006). In the late stages, the up-regulated NF-κB inhibitor and CD22 suggested remission, and the down-regulated HELLS (lymphoid-specific helicase) suggested reduced T cell proliferation (Geiman and Muegge, 2000). Yet, the up-regulated “phagosome” and “Jak-STAT signaling pathway” indicated some macrophage and T cell populations were still active. Additionally, C1q, C3, CFB, CFH and CFI, indicated alternative activation of the complement system (Lopez-Lera et al., 2018).

**Figure 8:**
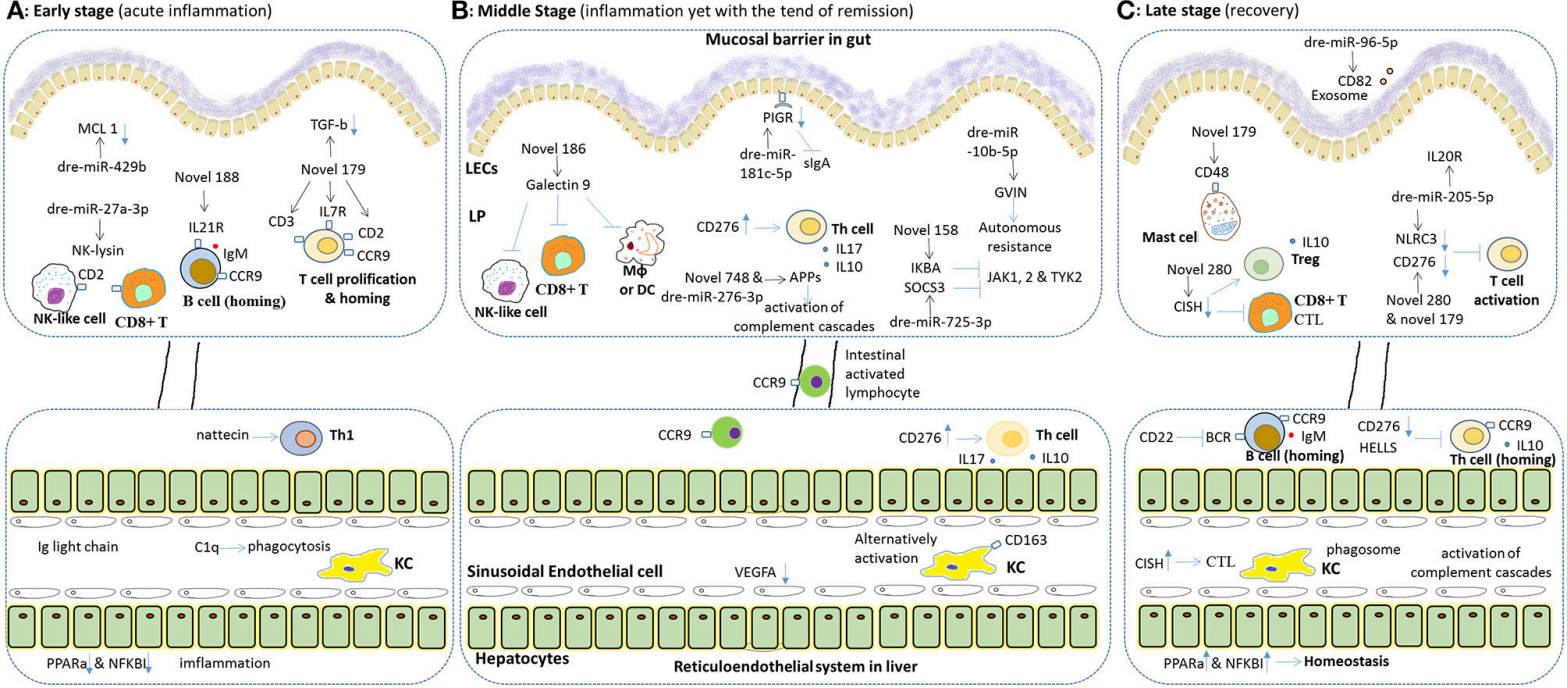
Hypothesis for immune mechanism for both the pathology and the resilience to SEM stress in fish gut and liver. Both intestinal and hepatic immune genes play important roles, in different stages of such resilience different immune reactions may exist. In general, the final result of the resilience to SEM in grass carp includes the recovery of the gut and relief in the liver, since the hepatic immune reaction may be later and longer. During the whole processes, intestinal miRNA played a key role on timely transcriptional regulation. **(A)** Upon on-setting of SBMIE, the intestinal lymphocytes, including T, B and NK cell, responded quickly, and epithelial cell began apoptosis (indicated by down-regulated MCL-1). In liver, phagocytosis and Th1 response may increase, while the accumulation of Ig light chain indicated tissue damage, and down-regulated PPARa & NKKBI indicated breaking of homeostasis. **(B)** In the middle stages, intestinal activation of T cell and complement cascades were the main reactions, whereas some enhancing of autonomous resistance was also found. Also, the B7 stimulated hepatic T cell response (e.g., secretion of IL17 and IL10) as well as the alternatively activating of hepatic macrophages was dominating. Besides, the intestinal activated lymphocytes could also be found in liver. **(C)** In the late stages, in gut, the enhancing gene expression related to Treg and mast cell indicated tolerance. In addition, IL20R signaling might indicate intestinal wound healing. Whereas, in the liver, inhibited T/B cell response and increased homeostasis related genes indicated remising, yet complement cascades as well as both IL10 and IgM still kept working. As to the predicted effect of immune genes, the “–|” means inhibition while the blue arrow means activation, and for the blue arrow with rectangle head, the up or down direction means the up- or down-regulated of gene expression. Besides, the effect of miRNA, the black arrow points out the immune mRNA target of each miRNA.

In conclusion, as the mechanism hypothesized in Figure 8, the resilience to SBM stress occurred in grass carp via enhancing intestinal immune tolerance and wound healing, paralleled with re-balancing hepatic immune responses. Our data systemically demonstrated the immune mechanism for the resilience to SBM stress in the fish gut-liver. Further effort will be made on functional studying on key intestinal or hepatic immune genes and miRNAs. The revealed resilience to SBM stress in herbivorous fish may help further study for oral tolerance of foodborne enteritis in other fish species.

## Author contributions

NW and Y-AZ conceived the projects. NW, BW, Z-WC, X-YZ, Z-XW, and D-DC performed the experiments. NW, BW, Y-YC, XX, and X-ML did data analysis. NW and BW wrote the manuscript. Y-AZ revised the manuscript.

### Conflict of interest statement

The authors declare that the research was conducted in the absence of any commercial or financial relationships that could be construed as a potential conflict of interest.
