# Supplementary material for: Integrative Transcriptomic and microRNAomic Profiling Reveals Immune Mechanism for the Resilience to Soybean Meal Stress in Fish Gut and Liver
Source: Front Physiol. 2018 Sep 10;9:1154. doi: 10.3389/fphys.2018.01154 (PMC6140834; doi:10.3389/fphys.2018.01154)
Supplement: Supplementary file 3 [file Table_3.DOC]

Table S3A Detailed information for quality of transcriptome data

| **Sample** | **Total reads** | **Total mapped** | **Multiple mapped** | **Uniquely mapped** | **Reads map to ‘+’** | **Reads map to ‘-’** | **Non-splice reads** | **Splice reads** |
| --- | --- | --- | --- | --- | --- | --- | --- | --- |
| Gut (0d)-1 | 47704952 | 39017850 (81.79%) | 1584418 (3.32%) | 37433432 (78.47%) | 18664299 (39.12%) | 18769133 (39.34%) | 18051176 (37.84%) | 19382256 (40.63%) |
| Gut (0d)-2 | 51142532 | 41972595 (82.07%) | 1504690 (2.94%) | 40467905 (79.13%) | 20191365 (39.48%) | 20276540 (39.65%) | 20704342 (40.48%) | 19763563 (38.64%) |
| Gut (0d)-3 | 44755806 | 37198394 (83.11%) | 1311320 (2.93%) | 35887074 (80.18%) | 17905226 (40.01%) | 17981848 (40.18%) | 17904355 (40%) | 17982719 (40.18%) |
| Gut (3w)-1 | 47505670 | 38971286 (82.04%) | 1702354 (3.58%) | 37268932 (78.45%) | 18586383 (39.12%) | 18682549 (39.33%) | 18875042 (39.73%) | 18393890 (38.72%) |
| Gut (3w)-2 | 49335270 | 40614547 (82.32%) | 1869448 (3.79%) | 38745099 (78.53%) | 19361412 (39.24%) | 19383687 (39.29%) | 20241389 (41.03%) | 18503710 (37.51%) |
| Gut (3w)-3 | 52603034 | 43453114 (82.61%) | 1990818 (3.78%) | 41462296 (78.82%) | 20687438 (39.33%) | 20774858 (39.49%) | 22347830 (42.48%) | 19114466 (36.34%) |
| Gut (5w)-1 | 49521272 | 40362100 (81.5%) | 1919409 (3.88%) | 38442691 (77.63%) | 19152246 (38.67%) | 19290445 (38.95%) | 20061382 (40.51%) | 18381309 (37.12%) |
| Gut (5w)-2 | 53392232 | 43704463 (81.86%) | 2233921 (4.18%) | 41470542 (77.67%) | 20662953 (38.7%) | 20807589 (38.97%) | 20964514 (39.27%) | 20506028 (38.41%) |
| Gut (5w)-3 | 48785908 | 40107377 (82.21%) | 1688899 (3.46%) | 38418478 (78.75%) | 19168895 (39.29%) | 19249583 (39.46%) | 19903700 (40.8%) | 18514778 (37.95%) |
| Gut (7w)-1 | 47326768 | 38209891 (80.74%) | 2017966 (4.26%) | 36191925 (76.47%) | 18029076 (38.09%) | 18162849 (38.38%) | 19654705 (41.53%) | 16537220 (34.94%) |
| Gut (7w)-2 | 50278206 | 41501999 (82.54%) | 2563292 (5.1%) | 38938707 (77.45%) | 19391461 (38.57%) | 19547246 (38.88%) | 18053800 (35.91%) | 20884907 (41.54%) |
| Gut (7w)-3 | 53989978 | 43789512 (81.11%) | 2209475 (4.09%) | 41580037 (77.01%) | 20716866 (38.37%) | 20863171 (38.64%) | 22506556 (41.69%) | 19073481 (35.33%) |
| Liver (0d)-1 | 44057652 | 36703239 (83.31%) | 1449387 (3.29%) ) | 35253852 (80.02%) | 17509281 (39.74%) | 17744571 (40.28%) | 17512253 (39.75%) | 17741599 (40.27%) |
| Liver (0d)-2 | 50625318 | 42852832 (84.65%) | 1409549 (2.78% | 41443283 (81.86%) | 20579465 (40.65%) | 20863818 (41.21%) | 19919129 (39.35%) | 21524154 (42.52%) |
| Liver (0d)-3 | 49078996 | 41398243 (84.35%) | 1471906 (3%) | 39926337 (81.35%) | 19902412 (40.55%) | 20023925 (40.8%) | 18396260 (37.48%) | 21530077 (43.87%) |
| Liver (3w)-1 | 46493580 | 39843674 (85.7%) | 1537490 (3.31%) | 38306184 (82.39%) | 19115292 (41.11%) | 19190892 (41.28%) | 16964147 (36.49%) | 21342037 (45.9%) |
| Liver (3w)-2 | 55483304 | 46555975 (83.91%) | 1978113 (3.57%) | 44577862 (80.34%) | 22240345 (40.08%) | 22337517 (40.26%) | 19794802 (35.68%) | 24783060 (44.67%) |
| Liver (3w)-3 | 48299960 | 41234229 (85.37%) | 1492056 (3.09%) | 39742173 (82.28%) | 19835507 (41.07%) | 19906666 (41.21%) | 17997689 (37.26%) | 21744484 (45.02%) |
| Liver (5w)-1 | 50705884 | 42983248 (84.77%) | 1902394 (3.75%) | 41080854 (81.02%) | 20474663 (40.38%) | 20606191 (40.64%) | 18589537 (36.66%) | 22491317 (44.36%) |
| Liver (5w)-2 | 44568634 | 37783158 (84.78%) | 1509355 (3.39%) | 36273803 (81.39%) | 18046271 (40.49%) | 18227532 (40.9%) | 16319338 (36.62%) | 19954465 (44.77%) |
| Liver (5w)-3 | 40816584 | 33967442 (83.22%) | 1355406 (3.32%) | 32612036  (79.9%) | 16231077 (39.77%) | 16380959 (40.13%) | 15797158 (38.7%) | 16814878 (41.2%) |
| Liver (7w)-1 | 48052480 | 40780357 (84.87%) | 1731242 (3.6%) | 39049115 (81.26%) | 19488278 (40.56%) | 19560837 (40.71%) | 17739450 (36.92%) | 21309665 (44.35%) |
| Liver (7w)-2 | 50525020 | 42507214 (84.13%) | 1668411 (3.3%) | 40838803 (80.83%) | 20397124 (40.37%) | 20441679 (40.46%) | 19276354 (38.15%) | 21562449 (42.68%) |
| Liver (7w)-3 | 54323858 | 46542621 (85.68%) | 2141848 (3.94%) | 44400773 (81.73%) | 22170615 (40.81%) | 22230158 (40.92%) | 19242176 (35.42%) | 25158597 (46.31%) |

Table S3B Detailed information for quality of small RNA data

| **Sample** | **Raw reads** | **Clean reads** | **Total reads of sRNA** | **Uniq reads of sRNA** | **Mapped sRNA** | **‘+’ mapped sRNA** | **‘-’ mapped sRNA** | **Mapped mature**  **(Upper: known;**  **Nether: novel)** | **Mapped hairpin**  **(Upper: known; Nether: novel)** | **Mapped star**  **(novel)** | **Mapped**  **uniq sRNA**  **(Upper: known;**  **Nether: novel)** | **Mapped**  **total sRNA**  **(Upper: known;**  **Nether: novel)** |
| --- | --- | --- | --- | --- | --- | --- | --- | --- | --- | --- | --- | --- |
| Gut (0d)-1 | 16724619 | 16583467 (99.16%) | 16438054 | 321707 | 14967647 (91.05%) | 6771764 (41.20%) | 8195883 (49.86%) | 256 | 254 | 99 | 3371 | 6920768 |
|  |  |  |  |  |  |  |  | 246 | 300 |  | 810 | 30963 |
| Gut (0d)-2 | 10924335 | 10519899 (96.30%) | 10370909 | 674076 | 8107697 (78.18%) | 3253522 (31.37%) | 4854175 (46.81%) | 248 | 251 | 63 | 2737 | 3243463 |
|  |  |  |  |  |  |  |  | 192 | 236 |  | 545 | 10319 |
| Gut (3w)-1 | 16474800 | 16344747 (99.21%) | 16187920 | 331936 | 14983249 (92.56%) | 7030121 (43.43%) | 7953128 (49.13%) | 260 | 294 | 103 | 3318 | 7764308 |
|  |  |  |  |  |  |  |  | 251 | 310 |  | 853 | 34020 |
| Gut (3w)-2 | 12190948 | 11776393 (96.60%) | 11661787 | 599441 | 9525536 (81.68%) | 4005003 (34.34%) | 5520533 (47.34%) | 251 | 270 | 58 | 2570 | 2810508 |
|  |  |  |  |  |  |  |  | 188 | 222 |  | 476 | 8939 |
| Gut (5w)-1 | 17588892 | 17469895 (99.32%) | 17147158 | 465664 | 15966186 (93.11%) | 7628252 (44.49%) | 8337934 (48.63%) | 262 | 259 | 124 | 3745 | 8380660 |
|  |  |  |  |  |  |  |  | 294 | 352 |  | 1101 | 38162 |
| Gut (5w)-2 | 12552038 | 12015100 (95.72%) | 11088994 | 1111591 | 8902446 (80.28%) | 4563118 (41.15%) | 4339328 (39.13%) | 255 | 290 | 78 | 2872 | 3840339 |
|  |  |  |  |  |  |  |  | 226 | 271 |  | 644 | 13084 |
| Gut (7w)-1 | 11231665 | 10818035 (96.32%) | 10635778 | 612683 | 8791007 (82.66%) | 4060236 (38.18%) | 4730771 (44.48%) | 257 | 275 | 85 | 3002 | 4616174 |
|  |  |  |  |  |  |  |  | 240 | 289 |  | 781 | 18099 |
| Gut (7w)-2 | 12548016 | 12437386 (99.12%) | 12146211 | 315671 | 11547686 (95.07%) | 5338510 (43.95%) | 6209176 (51.12%) | 256 | 270 | 90 | 3062 | 7386674 |
|  |  |  |  |  |  |  |  | 261 | 311 |  | 877 | 29715 |
