# Supplementary figures and images for: Integrative Transcriptomic and microRNAomic Profiling Reveals Immune Mechanism for the Resilience to Soybean Meal Stress in Fish Gut and Liver
Source: Front Physiol. 2018 Sep 10;9:1154. doi: 10.3389/fphys.2018.01154 (PMC6140834; doi:10.3389/fphys.2018.01154)

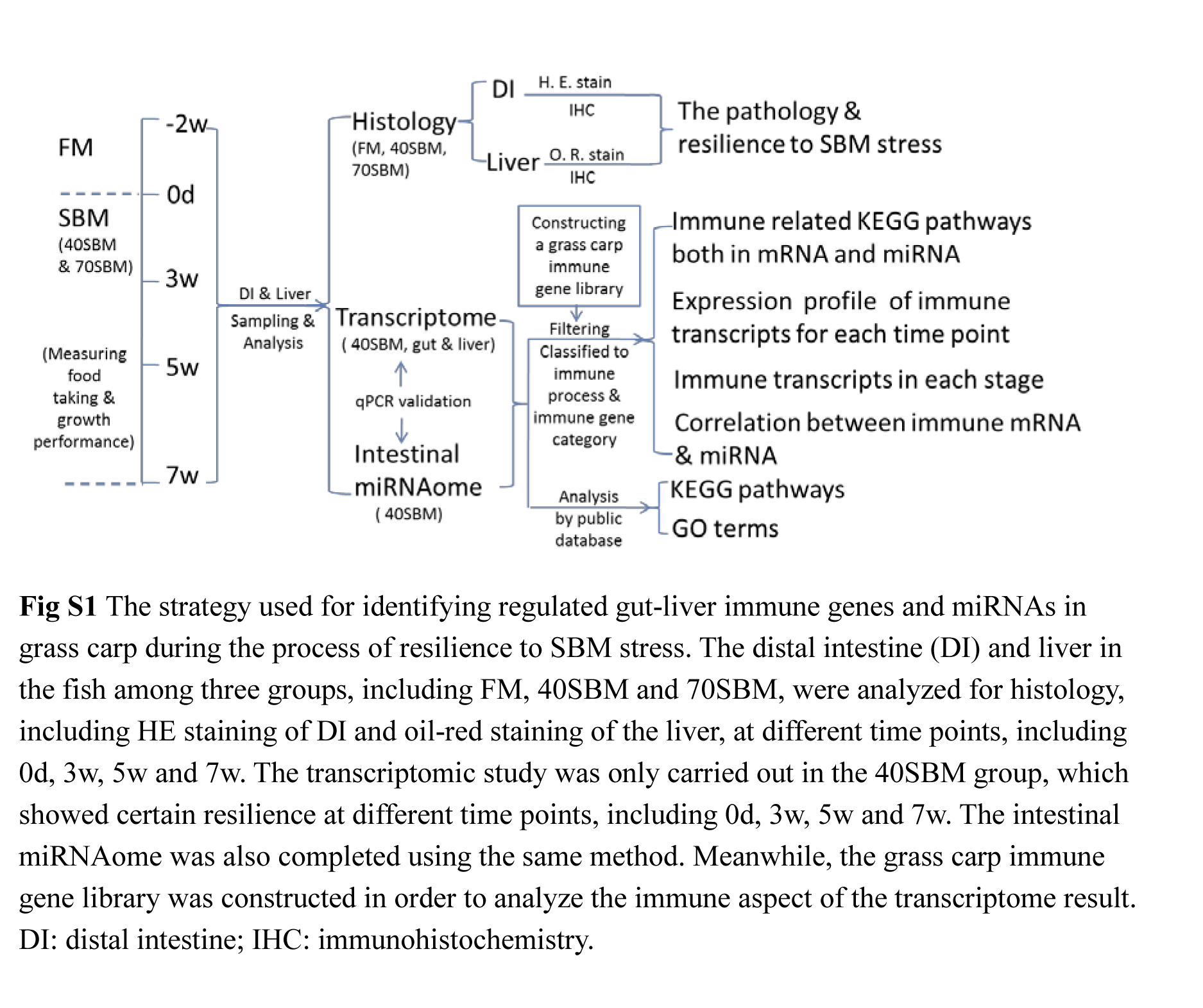

Supplement: Supplementary file 11 [file Image_1.TIF]

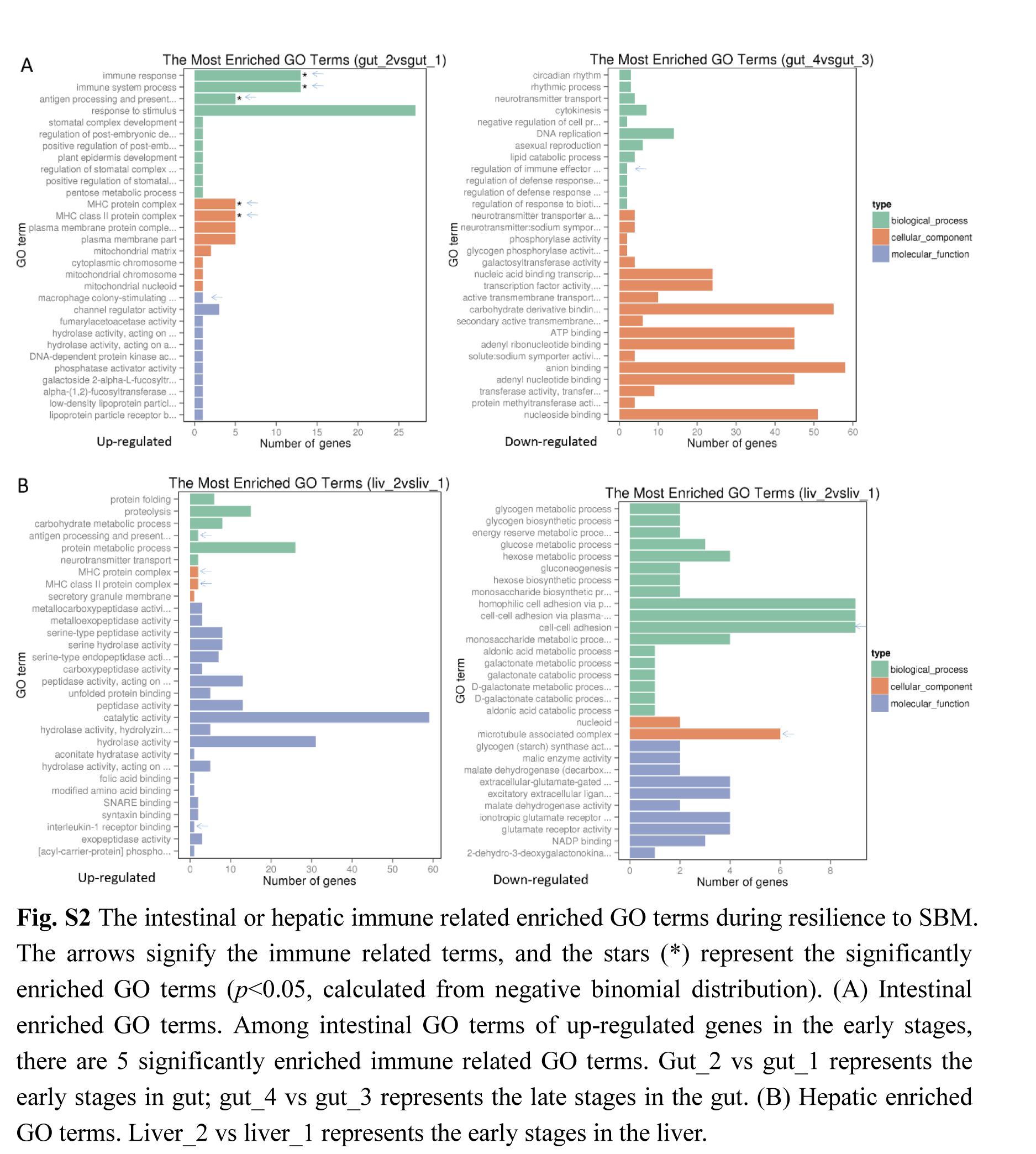

Supplement: Supplementary file 12 [file Image_2.TIF]

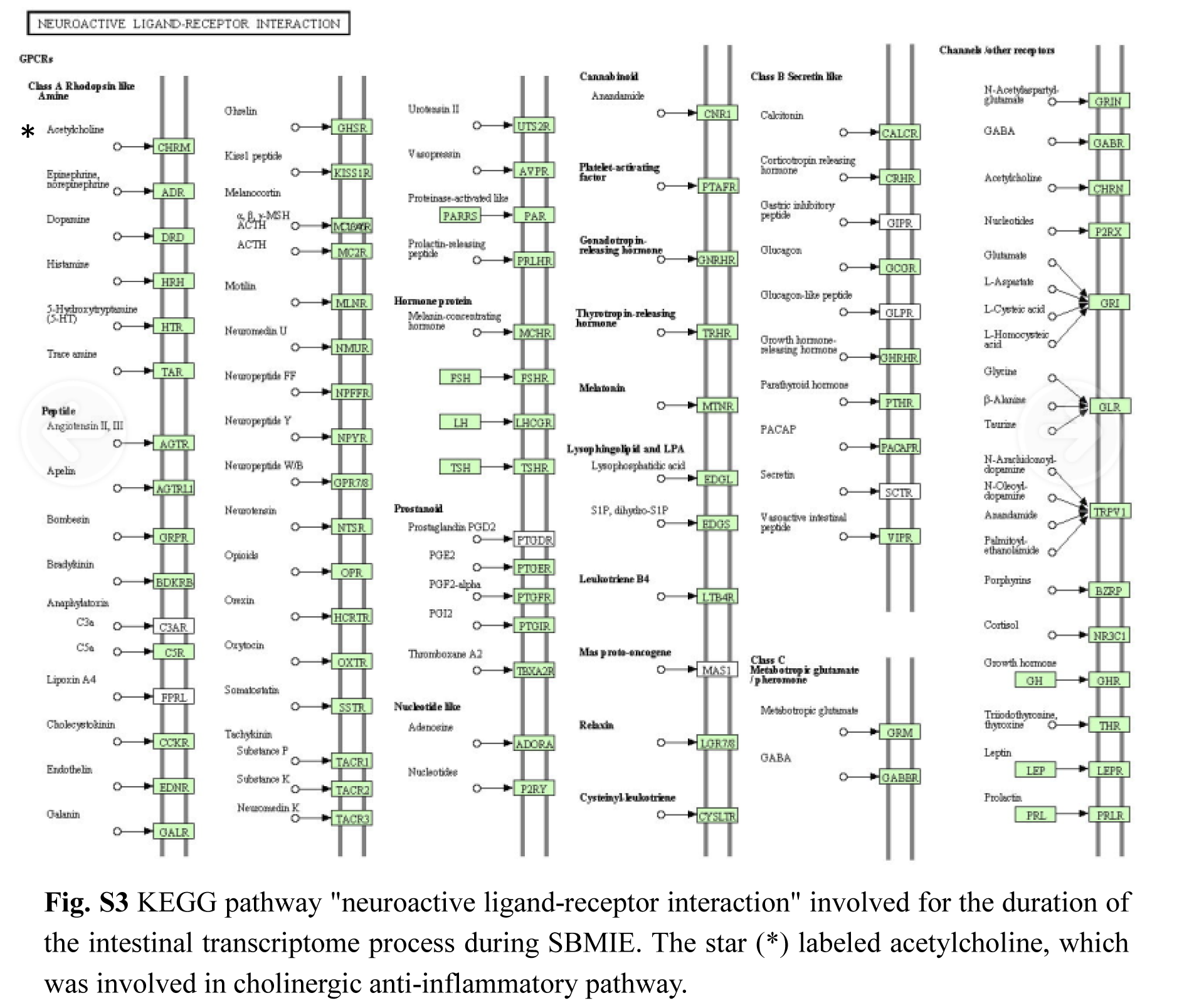

Supplement: Supplementary file 13 [file Image_3.TIF]
